# Protein Titration Benchmark of CHARMM36, Amber99sb-ildn, Amber14sb, OPLS-AA/M, and Martini 2 Force Fields for Constant pH Molecular Dynamics in GROMACS

**DOI:** 10.1021/acs.jctc.6c00678

**Published:** 2026-08-31

**Authors:** Noora Aho

**Affiliations:** † Computational Physics Laboratory, Tampere University, 33720 Tampere, Finland; ‡ Theoretical Physics and Center for Biophysics, Saarland University, 66123 Saarbrücken, Germany

## Abstract

Constant pH molecular dynamics (CpHMD) has emerged as a powerful method for simulating pH-dependent processes in proteins and other biomolecules. The original implementation of the PME-based CpHMD in the GROMACS software included the CHARMM36 and Martini 2 force fields for the treatment of the titratable amino acids in proteins. As requested by the user community, this work contains the parametrization of titratable residues for the Amber99sb-ildn, Amber14sb, and OPLS-AA/M force fields. In addition to carefully carrying out the parametrization process, the performance of the different force fields in the scope of CpHMD is evaluated by performing computational titrations for a set of benchmark proteins. The calculated p*K*_a_ values obtained using all force fields yield an average root-mean-square error of about 1 pH unit in comparison to experimental p*K*_a_ values, which is in line with previous CpHMD benchmarks. The presented work expands the applicability of the PME-based CpHMD method in GROMACS and guides the researchers aiming to perform accurate and efficient CpHMD simulations.

## Graphical abstract

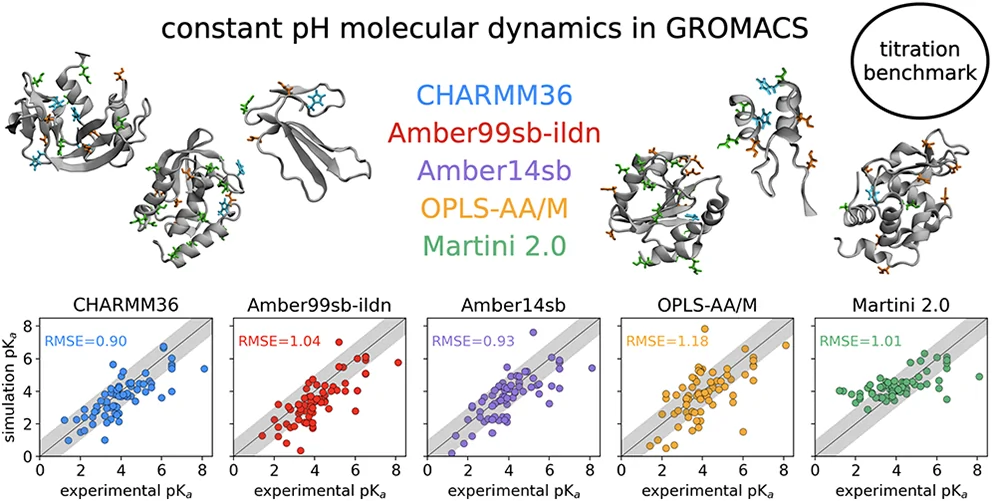

## Introduction

The protonation states of titratable, or ionizable, residues determine the overall charge state of a protein at a given pH. These residues, through their ability to gain or lose protons, play a central role in a wide range of biological processes. They are essential, for example, in enzyme catalysis, where proton transfer drives chemical reactions,^1,2^ in protein stability and folding,^3–5^ where salt bridges and hydrogen bonds depend on charge states, and in protein–ligand interactions, where electrostatic complementarity often relies on the protonation state of residues at binding interfaces.^6,7^

In addition to experimental techniques for probing the protonation of titratable residues, advances in constant pH molecular dynamics (CpHMD) have provided a computational approach for investigating pH-dependent processes in proteins and other biomolecules. A wide range of CpHMD implementations now exist across different molecular dynamics (MD) software packages, varying in how they represent protonation states (discrete or continuous), model solvent environments (implicit or explicit), and perform electrostatic calculations. To illustrate the broad applicability of CpHMD, recent studies have employed it to investigate protonation dynamics, for example, in protein–protein association,^8,9^ pH sensor proteins,^10,11^ enzyme active sites,^12,13^ and ligand permeation and binding.^14,15^

In discrete CpHMD methods, the free energy associated with changing a protonation state is estimated, and protonation transitions are performed discretely using a Monte Carlo scheme. In contrast, continuous CpHMD methods allow protonation states to evolve smoothly through the λ-dynamics approach.^16^ CpHMD methods can utilize either explicit water models, where each water molecule is represented individually at higher computational cost, or implicit solvent models, where the solvent is treated as a continuous low-dielectric medium, offering reduced computational expense. Most modern CpHMD implementations^17–20^ use the particle mesh Ewald (PME) method for calculating electrostatic interactions, although a recent implementation based on the fast multipole method (FMM) has also been developed.^21^ The most recent implementations of CpHMD include the PME-based continuous CpHMD with explicit water in GROMACS^17^ (used in this work), the PME-based continuous CpHMD with explicit water in CHARMM,^18^ the PME-based continuous CpHMD with explicit^19^ and implicit^22^ water in Amber, the discrete CpHMD with explicit water in NAMD,^20^ the discrete stochastic titration CpHMD with explicit water in GROMACS,^23^ and the FMM-based continuous CpHMD with explicit water in GROMACS.^21^

The original implementation of the PME-based CpHMD in GROMACS^17,24^ included the description of the titratable residues for the CHARMM36 ^25^ and Martini 2 ^26^ force fields. In order to expand the applicability of the GROMACS CpHMD, and as requested by the user community, this work adds the parametrization of titratable residues for the Amber99sb-ildn,^27^ Amber14sb,^28^ and OPLS-AA/M^29^ force fields. First, the parametrization of the titratable residues is validated by reproducing the correct titration behavior of the single titratable residues and reproducing their reference p*K*_a_ values in water. Second, the performance of the five force fields in the scope of CpHMD was evaluated by carrying out a protein titration benchmark. A set of six small proteins is titrated, and the calculated p*K*_a_ values are compared to experimental data. The inclusion of the Amber99sb-ildn, Amber14sb, and OPLS-AA/M as well as the presented protein titration benchmark broadens the applicability of the PME-based GROMACS CpHMD and gives the outline of parametrizing new titratable groups and performing accurate CpHMD simulations.

## Methods

This section describes the setup of the simulation systems and the parametrization of titratable residues for performing a protein titration benchmark using the various force fields with the GROMACS CpHMD method.^17,24,30^ All input files, including force fields, structures, topologies, simulation parameters, and scripts for setting up parametrization and titration, for reproducing the simulations can be found in Zenodo (10.5281/zenodo.17380681).

### Simulated Systems

To parametrize the titratable amino acids, the following Ala-X-Ala tripeptides were simulated: aspartic acid (Asp), glutamic acid (Glu), histidine (His), lysine (Lys), cysteine (Cys), and tyrosine (Tyr). Each tripeptide was placed in a periodic cubic box such that the distance from the tripeptide to the box edge was 1.5 nm, and filled with TIP3P^31^ water molecules. A 0.15 M concentration of Na+ and Cl– ions was added to all systems. To neutralize the system upon protonation state changes, each system contained 1 buffer particle^17,24^ in the parametrization simulations, and 5 buffer particles in the titration simulations. During the parametrization simulations, position restraints were applied to the CA atom of the titratable residue and the buffer particle.

Interactions were modeled using the CHARMM36,^25^ Amber99sb-ildn,^27^ Amber14sb,^28^ OPLS-AA/M,^29^ and Martini 2.0 ^26^ force fields. To accelerate the convergence of dihedral angles during CpHMD, small modifications to the dihedral potentials of titratable amino acids were introduced. For CHARMM36, these modifications are described in the GROMACS CpHMD publication,^24^ and for Amber99sb-ildn and Amber14sb, the modifications are described below. Titratable Asp, Glu, His, and Lys were included for all force fields, for CHARMM36 and Martini 2.0 already in our previous work,^17,24^ and for Amber99sb-ildn, Amber14sb, and OPLS-AA/M force fields in this work as described below. Titratable Cys and Tyr were included only for CHARMM36 (Cys parametrization done by Capelli et al.,^32^ Tyr in this work), Amber99sb-ildn, and Amber14sb, as for those force fields there were partial charges available for all protonation states.^23,33^

For the titration benchmark, the following water-soluble proteins were simulated: cardiotoxin V (cardV, PDB ID: 1CVO^34^), BBL (PDB ID: 1W4H^35^), hen egg white lyzozyme (HEWL, PDB ID: 2LZT^36^), Staphylococcal nuclease (SNase, PDB ID: 3BDC^37^), ribonuclease A (RNase A, PDB ID: 7RSA^38^), and thioredoxin (PDB ID: 1ERU^39^). All Asp, Glu, and His residues were made titratable, as experimental data exist for those residues in the selected proteins. Titration of Lys, Cys and Tyr residues in benchmark proteins was left for future work. Titration of the C- and N-termini was omitted, as parametrizing those for all the force fields used was beyond this work. Starting from the denoted .pdb files, structures and topologies compatible with GROMACS CpHMD were created using the phbuilder^40^ tool. Then, each protein was placed in a periodic cubic box such that the distance from the protein to the box edge was 2.0 nm, and filled with TIP3P^31^ (all-atom force fields) or polarizable (coarse-grained force field) water molecules. A 0.15 M concentration of Na^+^ and Cl^–^ ions was added to all protein systems. To neutralize the system upon protonation state changes, the total charge was constrained by adding 2*N*_site_ buffer particles,^24^ where *N*_site_ is the number of titratable residues in each system.

### Force Field Modifications for CpHMD

As observed for CHARMM36 and reported in the PME-based GROMACS CpHMD publication,^24^ the sampling of λ-coordinates is coupled to the sampling of certain dihedral angles in the side chains of titratable amino acids. To solve this, the barriers of side-chain dihedral potentials were lowered to ensure convergence of the dihedral angles and thus protonation states during CpHMD. The issue has been reported before for the carboxylic acid dihedral, and the CpHMD implementations for the Amber and CHARMM software generally increase the dihedral barrier, forcing the sampling of only the syn-conformation for Asp and Glu.^19,41,42^ Also in the recent implementation of fast multipole method (FMM) CpHMD in GROMACS, the syn orientation is enforced by the use of dihedral restraints.^21^ Following our previous approach described in Buslaev et al.^24^ for CHARMM36, the same dihedral modifications were introduced for Asp and Glu in Amber99sb-ildn and in Amber14sb, as a slow convergence of protonation states was observed. These modifications can be found in the force field and topology files provided in the SI. Dihedral angles for other residues (His, Lys, Cys, Tyr) for the Amber force fields, and for all residues for the OPLS-AA/M force field, converged within the simulation time, so the original force field was used.

In the Amber force field family, unlike CHARMM36 or OPLS-AA/M, the partial charges of the backbone are different for protonated and deprotonated amino acids. In order to keep the backbone charge unchanged during CpHMD for Amber99sb-ildn and in Amber14sb, the backbone charges of protonated residues were used, and the small residual charge for the deprotonated residue was absorbed into the C_β_ atom. This is the approach used by previous CpHMD implementations.^42^ All partial charges used can be found in the input files in SI.

### Parameterizing Titratable Residues

Prior to performing CpHMD simulations, the correction potential *V* ^MM^(λ) for each titratable residue needs to be calculated.^17,24^ The idea of *V* ^MM^(λ) is to compensate for the free energy difference between the protonation states for the titratable residue in water. The workflow of obtaining this correction potential is referred to as “parameterization” of a titratable residue for CpHMD. The coefficients for *V* ^MM^(λ) for Asp, Glu, His and Lys in CHARMM36 and Martini 2.0 can be found in the GROMACS CpHMD publications,^17,24^ and the workflow for the parametrization of residues for Amber99sb-ildn, Amber14sb and OPLS-AA/M contained the following steps: (1) calculation a rough estimate of the free energy of deprotonation using thermodynamic integration by linearly interpolating the λ-coordinate between λ = 0 and λ = 1, and fitting a polynomial to get the *V* ^MM^(λ) coefficients, (2) performing a set of so-called sampling simulations with CpHMD at pH = p*K*_a_ and zero barrier for the biasing potential, and plotting the distribution of the λ-coordinate, (3) in case of a nonflat distribution, updating the *V* ^MM^(λ) coefficients using the Boltzmann inversion of the distribution, and (4) performing a new set of sampling simulations with the updated coefficients and ensuring that the distributions are flat. This workflow is taken from the phbuilder^40,43^ publication.

In the scope of this work, the thermodynamic integration for two-state titratable residues (Asp, Glu, Lys, Cys, Tyr) was performed for λ = −0.1···1.1 with a step of 0.1, and each λ-value was simulated for 5 ns. The coefficients for *V* ^MM^(λ) (or precisely its derivative) were obtained using the fit_parameterization.py script from the phbuilder^40,43^ publication, fitting a fifth-order polynomial. Then, five replicas of 100 ns sampling simulations at pH = p*K*_a,ref_ and with zero barrier for the biasing potential were performed. After the introduced dihedral modifications, the λ-distributions from the five replicas were identical, and in the case of nonflat distributions, the *V* ^MM^(λ) coefficients were updated with the Boltzmann inversion, using the fit_parameterization.py script. Lastly, the updated coefficients were validated by performing another five replicas of 100 ns sampling simulations and observing flat distributions of the λ-coordinate. With the multisite representation (His), thermodynamic integration was performed by fixing the three λ-coordinates using the constraint λ_1_ + λ_2_ + λ_3_ = 1 and a step size of 0.1. With each set of λ-values on the grid, a 20 ns simulation was performed, after which the *V* ^MM^(λ) coefficients were fitted using a seventh-order polynomial. The coefficients were validated by performing five replicas of 100 ns sampling simulations. All coefficients for titratable residues Amber99sb-ildn, Amber14sb, and OPLS-AA/M can be found in the input files in the SI.

### Simulation Parameters

For the titration of the tripeptides, three replicas of 50 ns each were performed at 13 pH values, ranging from 1 to 7 for Asp and Glu, from 4 to 10 for His, from 8 to 14 for Lys, from 5 to 10 for Cys, and from 8 to 13 for Tyr. The reference p*K*_a_ values for tripeptides in water were 3.65, 4.25, 10.4, 6.42 (6.53, 6.94), 8.33, and 10.07 for Asp, Glu, Lys, His (HSD, HSE), Cys, and Tyr, respectively.^44–46^ For the proteins, three replicas of 100 ns each were performed at 21 pH values, ranging from 0.0 to 10.0. Other residues than Asp, Glu, and His in the proteins were not held titratable, as there was no corresponding experimental data. To estimate the p*K*_a_ values from the CpHMD simulations, for both tripeptides in water and for the titratable residues in proteins, the fraction of deprotonated frames was calculated at each pH, to which the Henderson–Hasselbalch equation was fitted. The details for the p*K*_a_ calculation can be found in the original GROMACS CpHMD publication.^17^ In addition, the SI contains plots for all residues with the Hill equation fitted to the fraction of deprotonated frames, including the fitted Hill coefficients.

#### All-Atom Force Fields

In all-atom simulations, the Coulomb interactions were modeled using the PME method with a real-space cutoff of 1.2 nm (CHARMM36) or 1.0 nm (Amber99sb-ildn, Amber14sb and OPLS-AA/M) and a grid spacing of 0.14 nm (CHARMM36), 0.125 (Amber99sb-ildn and Amber14sb) or 0.10 (OPLS-AA/M).^47,48^ For Lennard-Jones interactions, the forces were smoothly switched to zero in a range from 1.0 to 1.2 nm for CHARMM36, and the potentials were shifted by a constant such that they are zero at the cutoff of 1.0 nm for Amber99sb-ildn, Amber14sb, and OPLS-AA/M. For Amber99sb-ildn and Amber14sb, long-range dispersion correction for energy and pressure was applied. The temperature was kept constant at 300 K with the v-rescale thermostat^49^ using a coupling time of 0.5 ps, and the pressure was kept constant at 1 bar using the c-rescale barostat^50^ with a coupling time of 5.0 ps. The LINCS algorithm^51^ was used to constrain hydrogen bond lengths of the solutes, and the SETTLE^52^ algorithm was used to constrain the internal degrees of freedom of water molecules. Prior to the CpHMD simulations, the potential energy of each system was minimized using the steepest descent method, followed by 1 ns of equilibration. Specific for CpHMD, the mass of the λ-particles was set to 5 atomic units and the temperature was kept at 300 K with the v-rescale thermostat. The height of the biasing potential barrier was set to 5.0 kJ/mol for all titratable residues.

#### Martini

In the coarse-grained simulations with the Martini 2.0 force field, the Coulomb interactions were modeled using the reaction field potential with a 1.1 nm cutoff, a relative dielectric constant ϵ_*r*_ = 2.5, and a relative dielectric constant of the reaction field ϵ_RF_ = ∞. The Lennard-Jones interactions were truncated with a 1.1 nm cutoff, with the potentials shifted by a constant such that they are zero at the cutoff.^53^ The temperature was kept constant at 320 K with the v-rescale thermostat^49^ using a coupling time of 1.0 ps, and the pressure was kept constant at 1 bar using the Parrinello–Rahman barostat^54^ with a coupling time of 12.0 ps. The leapfrog integrator was used with an integration step of 20 fs. Prior to the CpHMD simulations, the potential energies of the systems were minimized using the steepest descent method, followed by 1 ns of equilibration. For CpHMD, the mass of the λ-particles was set to 10 atomic units and the temperature was kept at 300 K with the v-rescale thermostat. The height of the biasing potential barrier was set to 5.0 kJ/mol for all titratable residues.

## Results

Here, we report on the integration of the Amber99sb-ildn, Amber14sb, and OPLS-AA/M force fields into the PME-based GROMACS CpHMD framework^17^ and their evaluation in a protein titration benchmark. Our focus is on the force field modifications required for Amber99sb-ildn and Amber14sb, test titrations of the titratable residues in water, and the assessment of the accuracy and applicability of the different force fields for p*K*_a_ prediction across six benchmark proteins.

### Adding Titratable Residues for Amber99sb-ildn, Amber14sb, and OPLS-AA/M

During the parametrization of new titratable residues, where the correction potential *V* ^MM^ is determined, the need for force field modifications becomes apparent if the λ-distributions from sampling simulations differ between replicas. The sampling simulations are carried out at pH = p*K*_a,ref_ with a zero barrier for the biasing potential, under which a flat λ-distribution is expected. The upper panels of Figure 1 show the λ-distributions for the original and modified Amber99sb-ildn force field for aspartic acid (Asp) and glutamic acid (Glu). The same plots for Amber14sb are shown in Figure S2. With the original force field, the distributions vary across replicas, indicating convergence issues. As noted previously for GROMACS CpHMD,^24^ and as seen in the lower panels of Figure 1, this discrepancy can be attributed to differences in how replicas sample the syn- and anti-conformations of the carboxylic −COOH dihedral.

**Figure 1:**
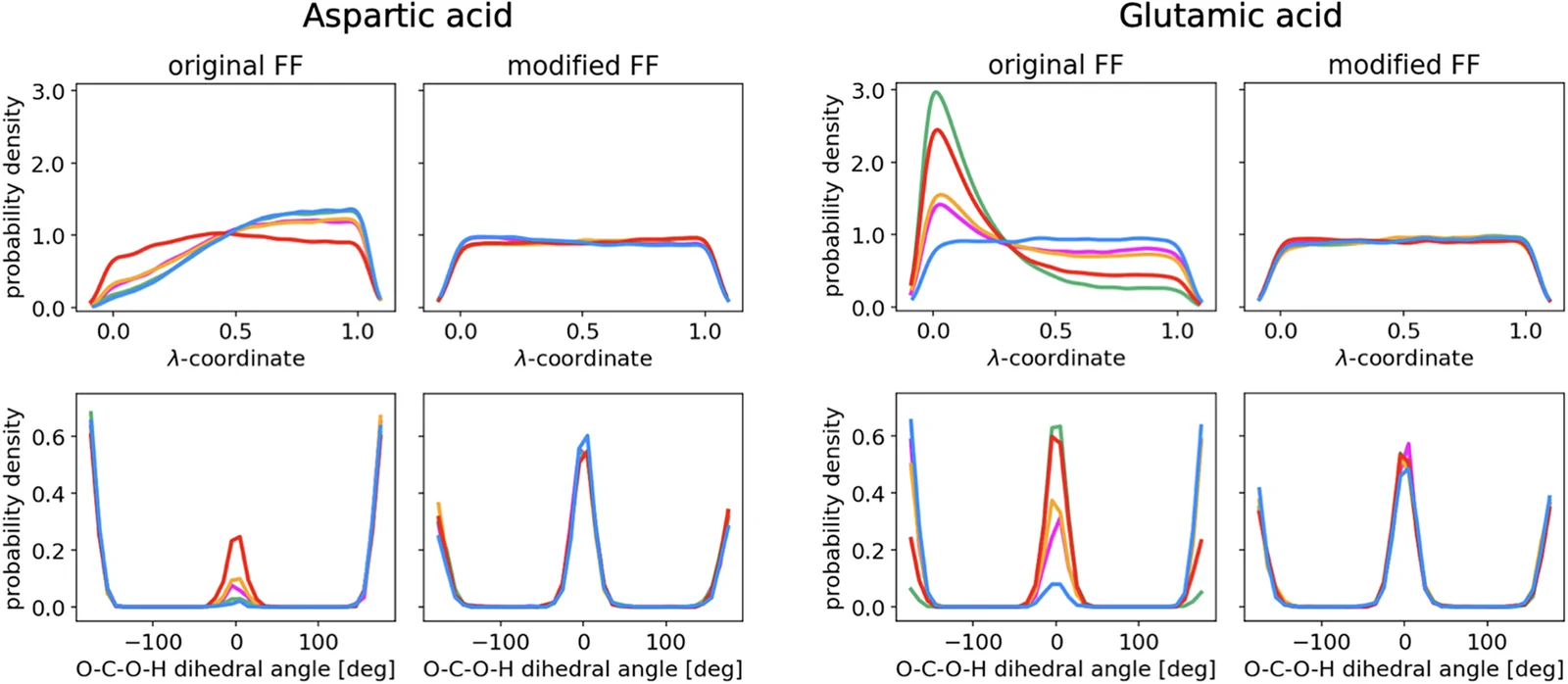
Sampling simulations of aspartic acid (left panels) and glutamic acid (right panels) in water using the original and modified Amber99sb-ildn force field. For both tripeptides, the distribution of the λ-coordinate (upper panels) and dihedral angle of the carboxylic group (lower panels) are shown. The sampling simulations are performed at pH = p*K*_a,ref_ and with zero barrier for the biasing potential, and the different colors represent the different replicas.

To improve sampling of the −COOH dihedral and thereby enhance convergence of the λ-distributions across replicas, we applied the same force field modifications for Asp and Glu in Amber99sb-ildn and Amber14sb as previously introduced for CHARMM36.^24^ These modifications lower the barrier of the −COOH dihedral potential, increasing transitions between the syn- and anti-conformations. An alternative strategy to improve convergence would be to restrict Asp and Glu to the syn-conformation, either by increasing the dihedral potential^19,41,42^ or by applying dihedral restraints.^21^ After lowering the dihedral potential, the −COOH dihedral distributions became consistent between replicas, and the λ-coordinate distributions were both uniform and reproducible across replicas, as shown in the right panels in Figure 1 (Amber99sb-ildn) and in Figure S2 (Amber14sb). The free energy profiles of the original and modified dihedral potentials for Asp and Glu in Amber99sb-ildn, obtained using the accelerated weight histogram (AWH) method, are shown in Figure S1. The modified parameters for Amber99sb-ildn and Amber14sb are provided in the input files as Supporting Information.

For histidine (His), lysine (Lys), cysteine (Cys), and tyrosine (Tyr), no force field modifications for Amber99sb-ildn or Amber14sb were required, and their λ-distributions are presented in Figure S3. For OPLS-AA/M, no modifications were necessary, as both the λ-coordinate and dihedral distributions were consistent across replicas. The flat λ-distributions for all titratable residues in water using OPLS-AA/M are also shown in Figure S3. Coefficients for the polynomial fit for the correction potential *V* ^MM^ for all titratable residues are given in the Supporting Information, both in the input files for phbuilder and in the corresponding .mdp files for CpHMD.

To validate the *V* ^MM^ coefficients for the newly parametrized titratable residues, computational titrations were performed for the tripeptides Ala–X–Ala in water. The resulting titration curves obtained with Amber99sb-ildn, Amber14sb, and OPLS-AA/M are shown in Figure 2, and the fitted p*K*_a,ref_ values are listed in Table 1. Corresponding results for CHARMM36 and Martini 2.0 can be found in the original publications of the PME-based GROMACS CpHMD.^17,24^

**Table 1:** Calculated p*K*_a_ Values for Asp, Glu, Lys, His, Cys and Tyrtripeptides in Water, using the Amber99sb-ildn, Amber14sb, and OPLS-AA/M Force Fields^a^

residue	Amber99sb-ildn	Amber14sb	OPLS-AA/M	refs 44–46
Asp	3.52 ± 0.06	3.57 ± 0.07	3.61 ± 0.05	3.65
Glu	4.13 ± 0.07	4.29 ± 0.06	4.23 ± 0.05	4.25
Lys	10.58 ± 0.06	10.61 ± 0.07	10.59 ± 0.06	10.40
His_macro_	6.41 ± 0.05	6.40 ± 0.09	6.43 ± 0.03	6.42
His_HSD_	6.60 ± 0.05	6.59 ± 0.06	6.58 ± 0.04	6.53
His_HSE_	7.01 ± 0.09	7.09 ± 0.20	7.04 ± 0.03	6.94
Cys	8.36 ± 0.06	8.37 ± 0.06	–	8.33
Tyr	10.01 ± 0.09	10.04 ± 0.05	–	10.07

a The reference values are given in the last column.^44–46^

**Figure 2:**
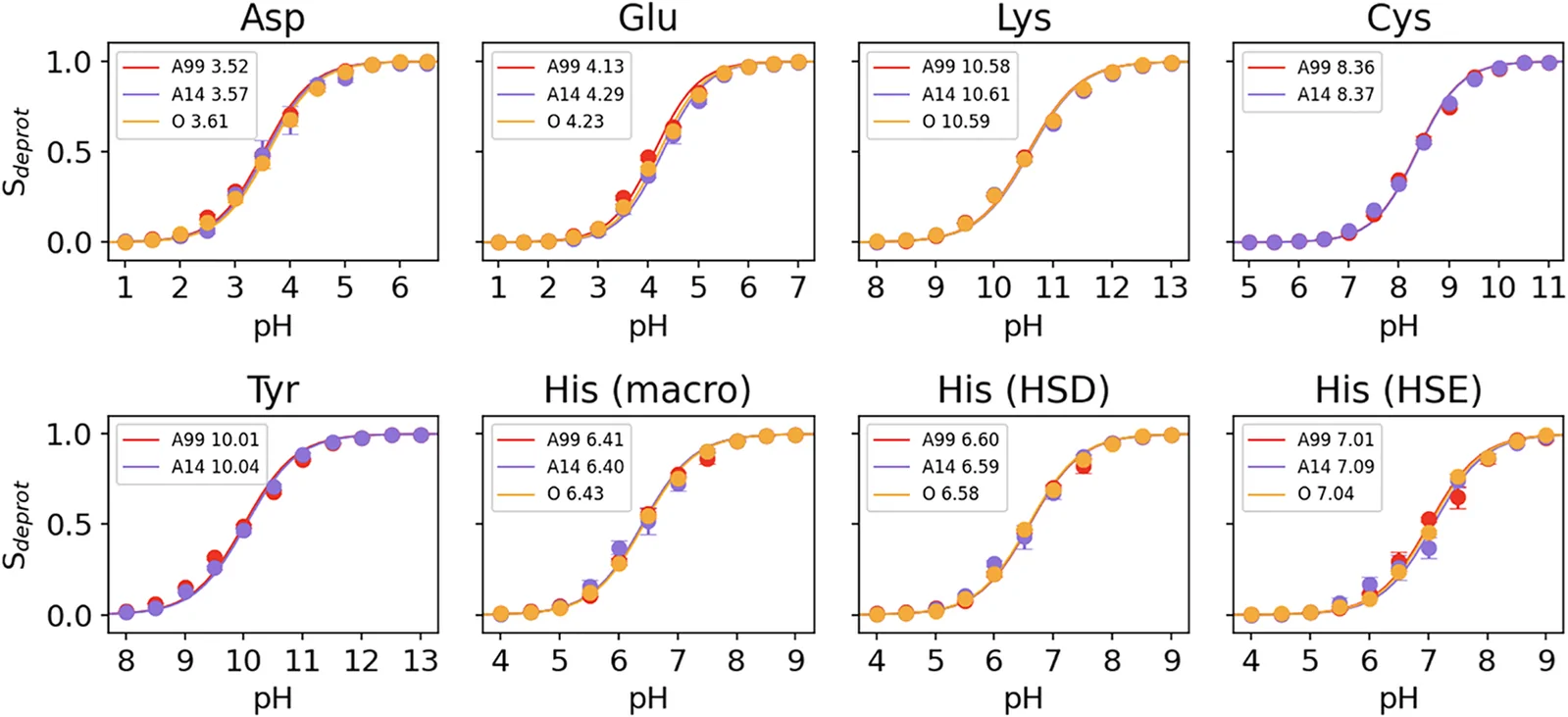
Titrations of tripeptide amino acids (Glu, Asp, Lys, Cys, Tyr, and His) in water, using the Amber99sb-ildn (A99), Amber14sb (A14), and OPLS-AA/M (O) force fields. Dots show the fraction of deprotonated acid, and the lines show the fits to the Henderson–Hasselbalch equation. The estimated p*K*_a_ values are given in the legends and in Table 1. For His, three separate titration curves for macroscopic and microscopic are shown, for the macroscopic (macro) and the microscopic (HSD and HSE) deprotonations. The errors were estimated as the standard error of the mean for the three replicas of 50 ns per pH point.

For Asp (p*K*_a,ref_ = 3.65), Amber99sb-ildn and Amber14sb predicted 3.52 ± 0.06 and 3.57 ± 0.07, respectively, while OPLS-AA/M gave 3.61 ± 0.05. Thus, the p*K*_a_ values predicted with Amber force fields are slightly less accurate but still very reasonable. Similarly for Glu (p*K*_a,ref_ = 4.25), Amber99sb-ildn and Amber14sb predicted 4.13 ± 0.07 and 4.29 ± 0.06, respectively, while OPLS-AA/M gave a slightly more accurate value of 4.23 ± 0.05. For His, Lys, Cys, and Tyr, all parametrized force fields performed equally well, with deviations of at most 0.3 units from the reference values.

### Protein Titration Benchmark

To validate the parametrization of titratable residues and to assess the performance of different force fields in estimating protein p*K*_a_ values, we carried out CpHMD titration simulations on a benchmark set of proteins. This benchmark involved calculating the p*K*_a_ values of all titratable Asp, Glu, and His residues in the following systems: cardiotoxin V (cardV), BBL, hen egg white lysozyme (HEWL), Staphylococcal nuclease (SNase), ribonuclease A (RNase A), and thioredoxin. These proteins were selected because they have previously been used as benchmarks for evaluating CpHMD implementations and force fields, and because experimentally determined p*K*_a_ values are available for comparison.^37,55–61^ Residues other than Asp, Glu, and His were not treated as titratable, as corresponding experimental data for the benchmark proteins were unavailable. Furthermore, Lys and Tyr typically have high p*K*_a_ values that lie outside the simulated pH range. Investigating the titration of Lys, Cys, and Tyr residues in specific proteins therefore remains a subject for future work, although the parametrization of this work provides the framework to enable such studies.

Figure 3 compares the calculated and experimental p*K*_a_ values for the titratable residues (Asp, Glu, and His) in the six benchmark proteins using the CHARMM36, Amber99sb-ildn, Amber14sb, OPLS-AA/M, and Martini 2.0 force fields. The structures of the proteins are shown above each plot, with titratable residues highlighted. Complete titration curves for all residues are provided in Supporting Figures S4–S9, and the full set of calculated and experimental p*K*_a_ values with associated errors are reported in Tables S1–S6. Overall, the calculated p*K*_a_ values agree reasonably well with experiment, with root-mean-square error (RMSE) typically ranging from 0.7 to 1.3 across most proteins and force fields. Importantly, the RMSE varies across proteins and force fields, and no single force field consistently outperforms the others. For each protein, outlier residues are observed where the CpHMD-predicted p*K*_a_ deviates substantially from the experimental value, and the residues showing this deviation differ among force fields.

**Figure 3:**
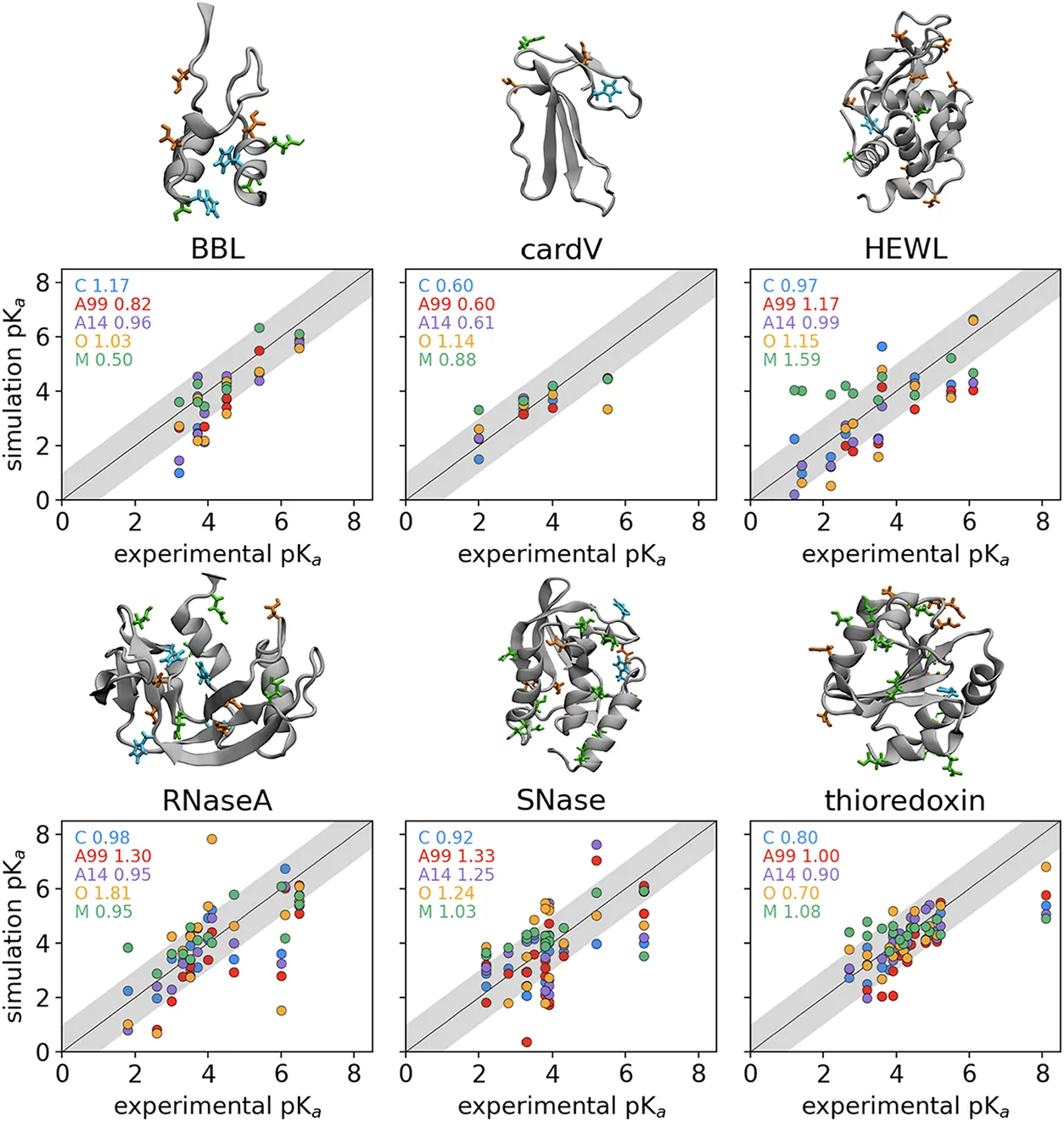
Comparison between calculated and experimental p*K*_a_ values for the titratable residues (Asp, Glu, His) in the six benchmark proteins using the CHARMM36, Amber99sb-ildn, Amber14sb, OPLS-AA/M, and Martini 2.0 force fields. For each protein, the corresponding structure is shown above the plot, with titratable residues highlighted as sticks (Asp in orange, Glu in green, and His in blue). The root-mean-square error (RMSE) for each force field is reported in the legend of the respective plot.

To assess the accuracy of different force fields in predicting p*K*_a_ values, Figure 4 summarizes the results for all benchmark proteins. The overall RMSE values are 0.90, 1.04, 0.93, 1.18, and 1.01 for CHARMM36, Amber99sb-ildn, Amber14sb, OPLS-AA/M, and Martini 2.0, respectively. As seen in Figure 4, despite its moderate overall RMSE of 1.01, Martini 2.0 systematically fails to reproduce the large downshifts in p*K*_a_ observed for certain residues. This limitation likely arises from the reduced accuracy of electrostatic interactions in the coarse-grained descriptions. In Martini 2.0, several atoms form one bead, hydrogen atoms disappear, and hydrogen bonds are encoded only implicitly. This could reduce the stability of the deprotonated state, failing to reproduce the large p*K*_a_ downshifts. Improvements in electrostatics, and therefore in p*K*_a_ predictions, could be achieved with the more recent Martini 3 force field.^62^ However, because a parametrization of the polarizable water model is not yet available for Martini 3, such simulations and the further analysis of using the Martini force field with CpHMD remain as tasks for future work.

**Figure 4:**
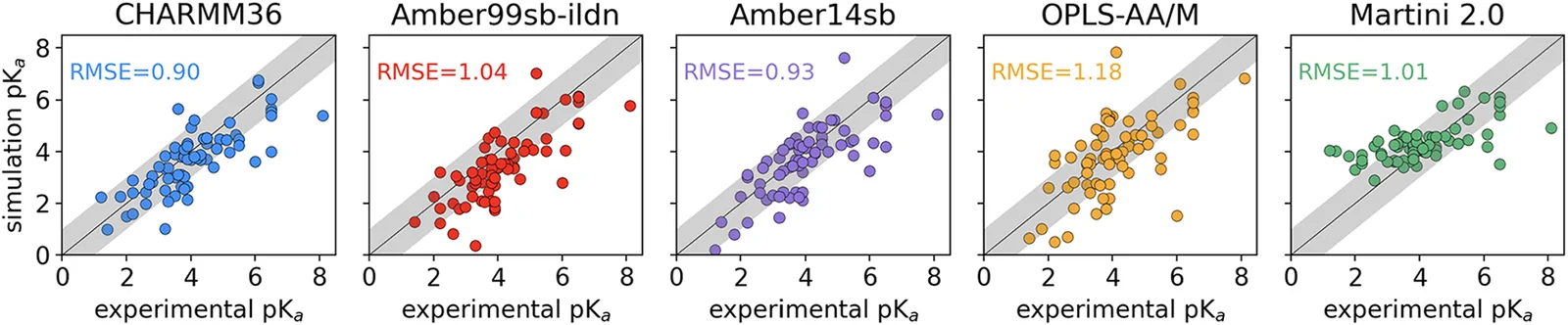
Comparison of calculated and experimental p*K*_a_ values across different force fields (CHARMM36, Amber99sb-ildn, Amber14sb, OPLS-AA/M, and Martini 2.0). Each plot includes the p*K*_a_ values for titratable residues (Asp, Glu, His) in all six benchmark proteins, with the overall root-mean-square error (RMSE) reported in the legend.

In this work, we do not examine every titratable residue with deviations from experimental p*K*_a_ values in detail but mention a few representative cases that have also been highlighted in previous CpHMD works.^18,19,63^ For BBL, the RMSE obtained with CHARMM36 is 1.17, which is slightly higher than that of the other force fields. A major contributor to this deviation is Asp-162, for which CHARMM36 predicts a p*K*_a_ of 1.0, compared with the experimental value of 3.2 (Figure S3 and Table S1). Amber99sb-ildn predicts a much closer p*K*_a_ of 2.65 for Asp-162 compared to the experimental value, whereas Amber14sb also underestimates it with a p*K*_a_ of 1.45. A too low p*K*_a_ for Asp-162 using Amber14 has also been reported previously,^63^ possibly due to overly stable hydrogen bonding with nearby residues.

For cardV and RNase A proteins, the largest RMSEs are observed with OPLS-AA/M, being 1.14 and 1.81, respectively. For cardV, the higher RMSE is driven primarily by the too low predicted p*K*_a_ of 3.34 for His-4 (Figure S5 and Table S2). Across all force fields, the titration curves of His-4 show slower convergence relative to other residues within the simulated time scale, suggesting slowly equilibrating interactions with neighboring residues. For RNase A, the RMSE obtained with OPLS-AA/M is 1.81, notably higher than that for the other all-atom force fields. Two contributors are Glu-86, for which OPLS-AA/M predicts a substantially elevated p*K*_a_, and His-12, for which the predicted p*K*_a_ is too low (Figure S7 and Table S4). The titration curves for both of these residues seem not to converge well in the simulation time (Figure S6), suggesting interactions with the neighboring residues. Buried or strongly interacting residues would need longer sampling times and further investigation, which is beyond the scope of this benchmark.

For SNase, the most challenging residues are Asp-19 and Asp-20, whose titration has been found to be coupled through a hydrogen-bond interaction.^18,19^ In our benchmark, OPLS-AA/M overestimates the p*K*_a_ of Asp-19, while the titration curve of Asp-20 fails to converge within the simulated time scale. The other all-atom force fields reproduce the coupled titration more reliably (Figure S8 and Table S5). OPLS-AA/M also overestimates the p*K*_a_ values of several Glu residues, including Glu-101, Glu-122, and Glu-129. In thioredoxin, the upshifted p*K*_a_ values of the important buried residues Asp-26 (p*K*_a_ = 8.1) and Asp-58 (p*K*_a_ = 5.2) are qualitatively reproduced, but all force fields underestimate the magnitude of the shift for Asp-26, contributing to the RMSE. Reproducing the p*K*_a_ values for these residues has also been challenging in previous work.^19^

To further investigate differences in p*K*_a_ prediction accuracy across residue types, Figure 5 compares the calculated and experimental p*K*_a_ values for Asp, Glu, and His residues. The results indicate that the force fields perform similarly for Asp and His residues, with no substantial differences in prediction accuracy. In contrast, CHARMM36 provides the most accurate predictions for Glu residues, achieving the lowest RMSE (0.52) among all of the force fields. Yet, large p*K*_a_ shifts remain difficult to reproduce consistently across all force fields, as they are often associated with coupled residue interactions that converge slowly during the simulations.

**Figure 5:**
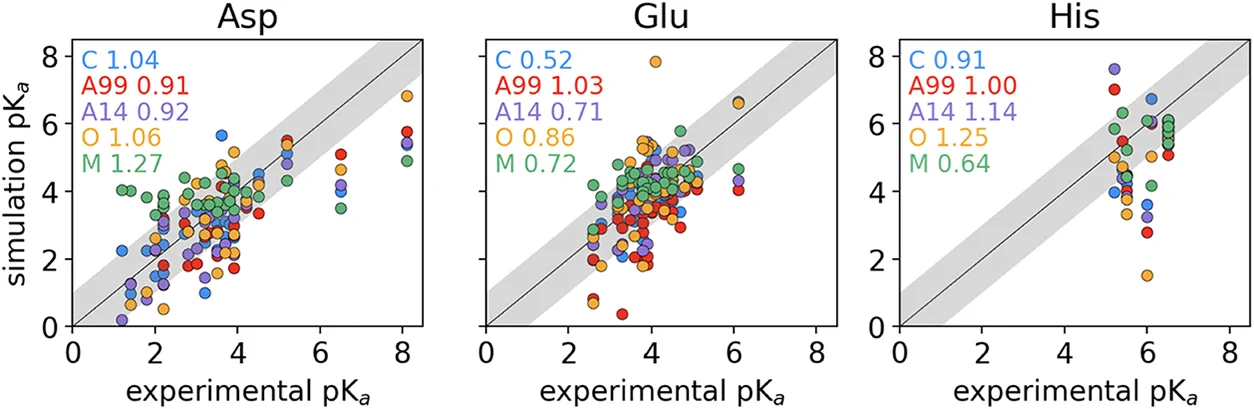
Comparison of calculated and experimental p*K*_a_ values across different force fields (CHARMM36, Amber99sb-ildn, Amber14sb, OPLS-AA/M, and Martini 2.0), divided into separate plots by titratable residue type. Each plot includes the p*K*_a_ values for either Asp, Glu, or His, with the overall root-mean-square error (RMSE) for all six benchmark proteins for different force fields reported in the legend.

Overall, with the exception of Martini 2.0, all force fields reproduce p*K*_a_ shifts in the benchmark proteins with reasonable accuracy apart from a few challenging residues. This suggests that all of them are generally suitable for performing GROMACS CpHMD simulations to study proteins in which protonation states and pH are coupled to conformational changes. At the same time, no single force field emerges as consistently superior since each shows deviations from experimental p*K*_a_ values for different titratable residues. Since no single force field consistently outperforms the others and each one shows deviations from experimental p*K*_a_ values for different residues, no recommendations for a force field as the most accurate choice for modeling protonation in proteins with CpHMD can be made. The choice of the most suitable force field is left to the user, depending on the system of interest. If possible, then we recommend always testing multiple force fields. As seen by the few titratable residues whose calculated p*K*_a_ values show large errors, the convergence of the deprotonation fractions with time should be monitored closely, especially when investigating tightly interacting or buried residues.

## Conclusions

The original PME-based GROMACS CpHMD implementation included titratable residue descriptions for the CHARMM36 and Martini 2 force fields. In this work, we extend this functionality by adding titratable Asp, Glu, His, Lys, Cys, and Tyr residues to the Amber99sb-ildn, Amber14sb, and OPLS-AA/M force fields, thereby making them compatible with the GROMACS CpHMD method. We provide a detailed description of the parametrization workflow and discuss required dihedral modifications for the titratable residues, offering practical guidance for users who wish to include additional titratable groups of interest to the CpHMD method. To validate the newly added titratable sites and compare the performance of different force fields within the CpHMD framework, we conducted titration benchmarks for six small proteins. The tested all-atom force fields yield an average root-mean-square error of approximately 1 between calculated and experimental p*K*_a_ values, consistent with previous CpHMD benchmarks. In contrast, we saw that the coarse-grained Martini 2.0 force field fails to reproduce large downshifts in p*K*_a_ values, otherwise being as accurate as all-atom forces.

It should be emphasized, however, that the ability to precisely reproduce experimental p*K*_a_ values should not be the sole motivation or objective for developing CpHMD methods. CpHMD implementations that demonstrate reasonable accuracy in capturing protonation dynamics and p*K*_a_ shifts can be applied to provide valuable insights into pH-dependent phenomena in proteins and other biomolecules. They offer a powerful tool to investigate, for example, structural changes, interactions, and binding energetics under varying pH conditions. We hope that this benchmark work broadens the applicability of the GROMACS CpHMD implementation and encourages researchers to incorporate pH effects into their biomolecular MD simulations.

## Supplementary Material



## Notes

MD simulations in the presented work in Zenodo (10.5281/zenodo.17380681); dihedral potentials calculated with AWH and λ-distributions for the single titratable residues in water using Amber99sb-ildn, Amber14sb, and OPLS-AA/M; titration curves and p*K*_a_ values for all titratable residues in all proteins using the five different force fields (PDF).
