# Supplementary material for: Protein Titration Benchmark of CHARMM36, Amber99sb-ildn, Amber14sb, OPLS-AA/M, and Martini 2 Force Fields for Constant pH Molecular Dynamics in GROMACS
Source: J Chem Theory Comput. 2026 Aug 31;22(18):9548–56. doi: 10.1021/acs.jctc.6c00678 (PMC13602905; doi:10.1021/acs.jctc.6c00678)
Supplement: Supplementary file 1 [file ct6c00678_si_001.pdf]

Supplementary Information:

Protein titration benchmark of CHARMM36, Amber99sb-ildn, Amber14sb, OPLS-AA/M, and Martini 2 force fields for constant pH molecular dynamics in GROMACS

Noora Aho*,†

†*Computational Physics Laboratory, Tampere University, 33720 Tampere, Finland*

‡*Theoretical Physics and Center for Biophysics, Saarland University, 66123 Saarbrücken, Germany*

E-mail: noora.aho@tuni.fi

1 Parameterizing titratable residues for Amber99sb-ildn, Amber-14sb and OPLS-AA/M

For the Amber99sb-ildn and Amber14sb force field, the dihedral potentials of the Asp and Glu sidechains were modified, in order to lower the barrier of the slowly converging -COOH angle. Dihedrals in Lys, Cys, His and Tyr were not modified, as all dihedral angles converged within simulation time. These modifications are the same as the ones previously defined for the CHARMM36 force field.[1] For OPLS-AA/M, no modifications were needed, as all dihedral angles converged within simulation time.

To quantify the difference between the dihedral potentials of the original and modified Amber99sb-ildn force field (the same modifications were applied for Amber14sb), we calculated the dihedral potential energy for the sidechain dihedrals for protonated and deprotonated Asp and Glu using the accelerated weight histogram method (AWH)[2] in GROMACS.[3] The input parameters for the AWH calculations can be found in the simulations files provided in `doi.org/10.5281/zenodo.17380682`. Dihedral potentials for N-CA-CB-GG, CA-CB-CG-OD2 and OD1-CG-OD2-HD2 in Asp are shown in the upper panel of Figure S1,

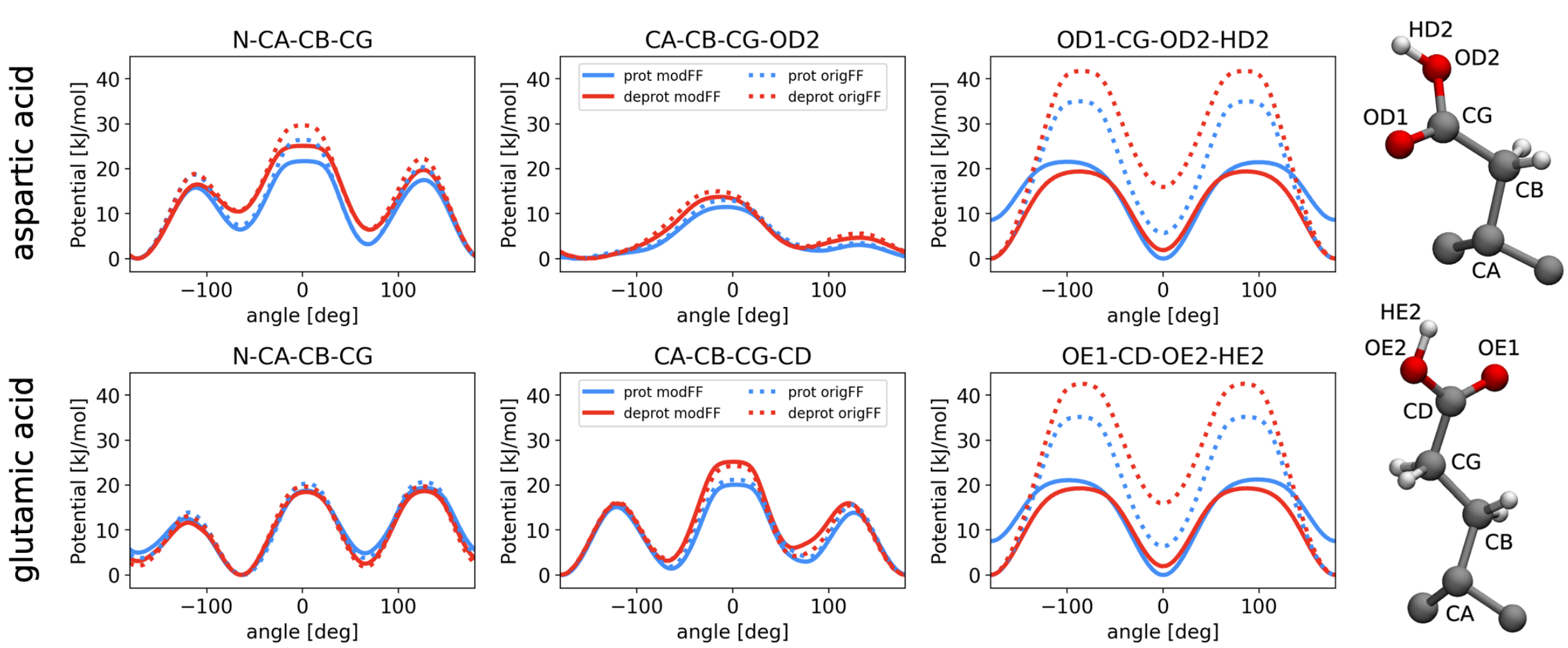

Figure S1: Dihedral potentials for Asp (upper row) and Glu (lower row) calculated using AWH, for both protonated (H+) and deprotonated (H–) states with the original and modified Amber99sb-ildn force field. The atomic nomenclature for Asp and Glu is shown on the right.

and the ones for N-CA-CB-GG, CA-CB-CG-CD and OE1-CD-OE2-HE2 in Glu in the lower panel of Figure S1. For both amino acids, the barrier of the carboxylic dihedral (OD1-CG-OD2-HD2 or OE1-CD-OE2-HE2) was lowered with the force field modifications, improving the sampling of the syn- and anti-conformations.

Figure S2 shows the distributions of the λ-coordinates and dihedral angles for Asp and Glu using the Amber14sb force field. Figure S3 shows the distributions of the λ-coordinates for the sampling simulations (pH=pK_a, zero barrier for the biasing potential) of Asp, Glu, His, Lys, Cys and Tyr in water (Cys and Tyr were not parameterized for OPLS-AA/M, hence missing in the figure). As the distributions are flat and the five replicas of 100 ns consistent, the protonation behaviour of the single titratable residues in water is as intended.

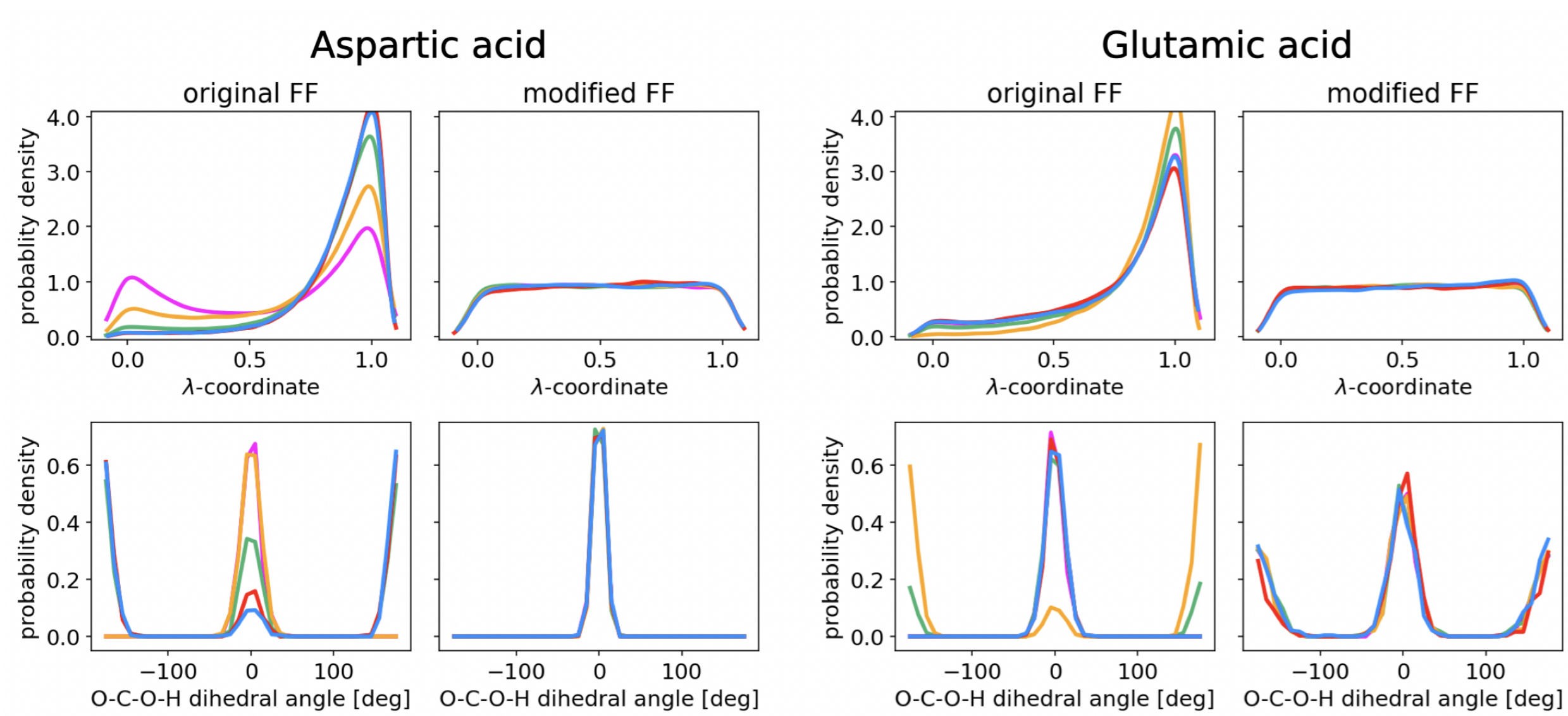

Figure S2: Sampling simulations of Asp (left panels) and Glu (right panels) in water using the original and modified Amber14sb force field. For both tripeptides, the distribution of the λ-coordinate (upper panels) and dihedral angle of the carboxylic group (lower panels) are shown. The sampling simulations are performed at pH=p$K_{a,ref}$ and with zero barrier for the biasing potential, and the different colors represent the different replicas.

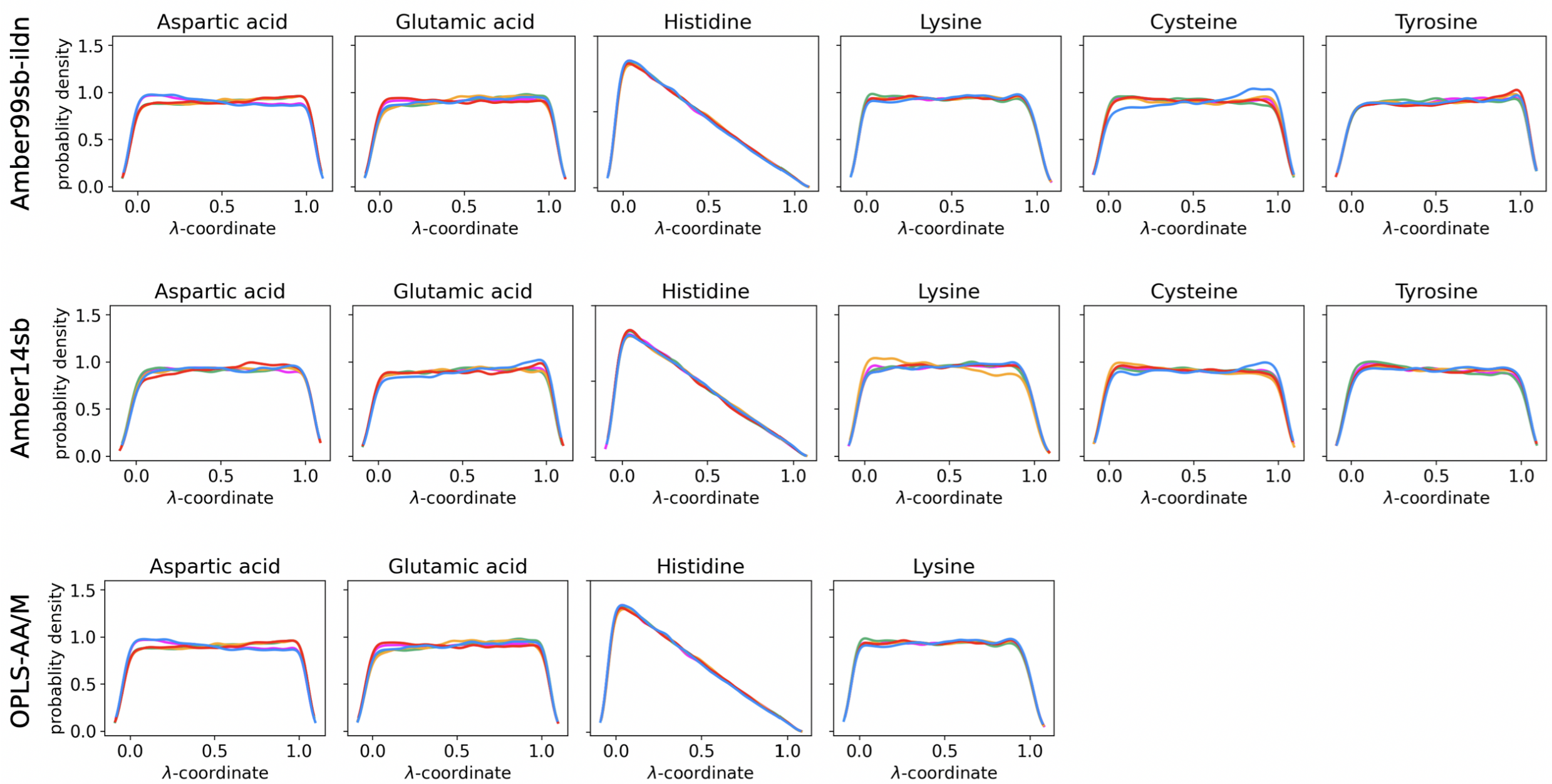

Figure S3: Distribution of the λ-coordinate for the sampling simulations of titratable residues (Asp, Glu, His, Lys, Cys, Tyr) for the modified Amber99sb-ildn, modified Amber14sb, and the original OPLS-AA/M force fields. The sampling simulations are performed at pH=p$K_{\mathrm{a,ref}}$ and with zero barrier for the biasing potential, and the different colors represent the different replicas.

2 Protein titration benchmark

The titration curves for all titratable residues in all benchmark proteins obtained using the different force fields are plotted in Figures S4–S9. The dots show the fraction of deprotonated acid, and the lines show the fits to the Henderson–Hasselbalch equation. The colors define the force field used, CHARMM36 in blue, Amber99sb-ildn in red, Amber14sb in purple, OPLS-AA/M in yellow, and Martini 2.0 in green. The estimated pK_a values are given in the legends, where the letter refers to the first letter of the force field. The experimentally obtained pK_a values,[4–11] if available and larger than zero, are illustrated with the vertical black lines. All estimated pK_a values with errors from the CpHMD simulations, in comparison to the experimental ones for all titratable residues using the different force fields, are listed in Tables S1–S6.

In addition to fitting the fraction of deprotonated acid to the Henderson-Hasselbalch equation, we also provide the fits to the Hill (generalized Henderson–Hasselbalch) equation. These plots along with the Hill coefficients can be found in Figures S10–S15.

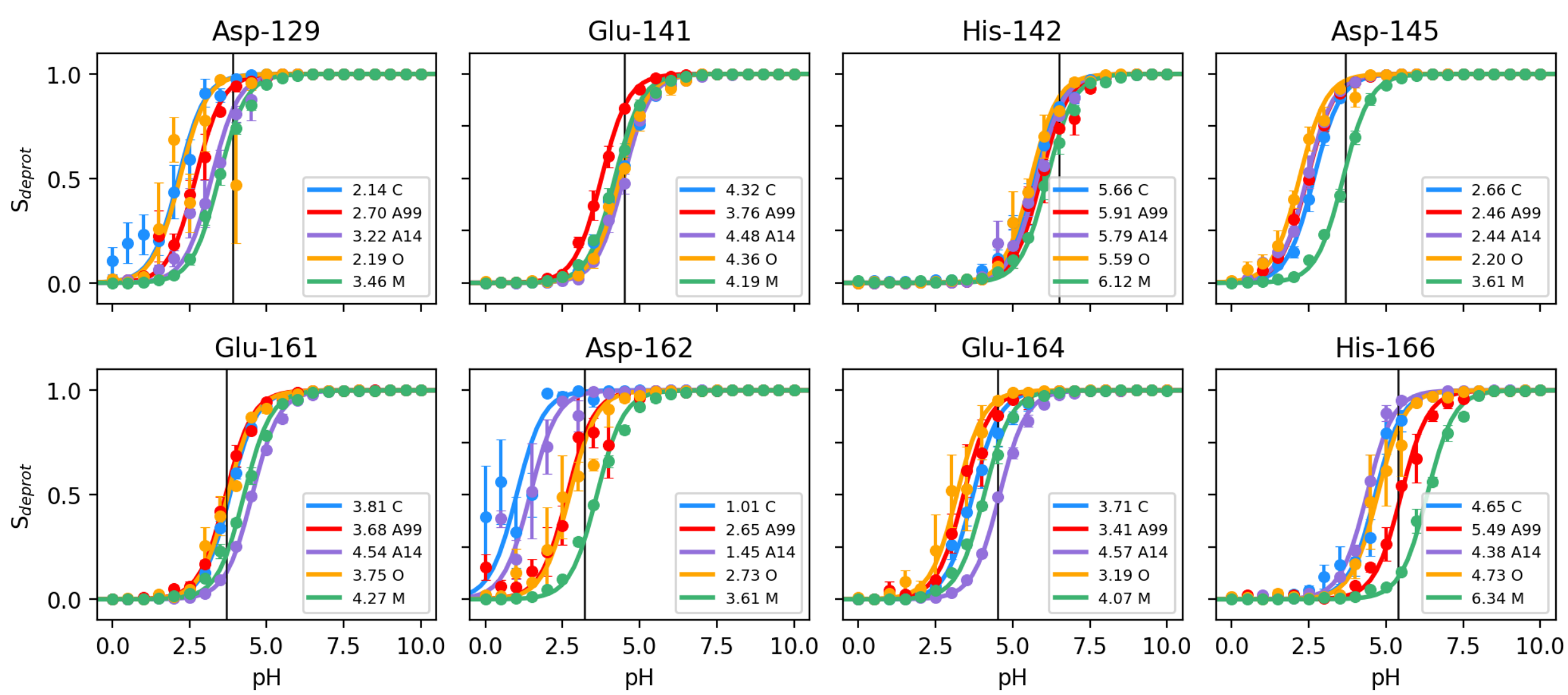

Figure S4: Titration curves for all titratable Asp, Glu and His in the BBL protein, using the CHARMM36 (blue), Amber99sb-ildn (red), Amber14sb (purple), OPLS-AA/M (yellow), and Martini 2.0 (green) force fields. The experimentally obtained pK_a values[4,5] are illustrated with vertical black lines. The estimated pK_a values are given in the legends, and the values with errors are listed in Table S1.

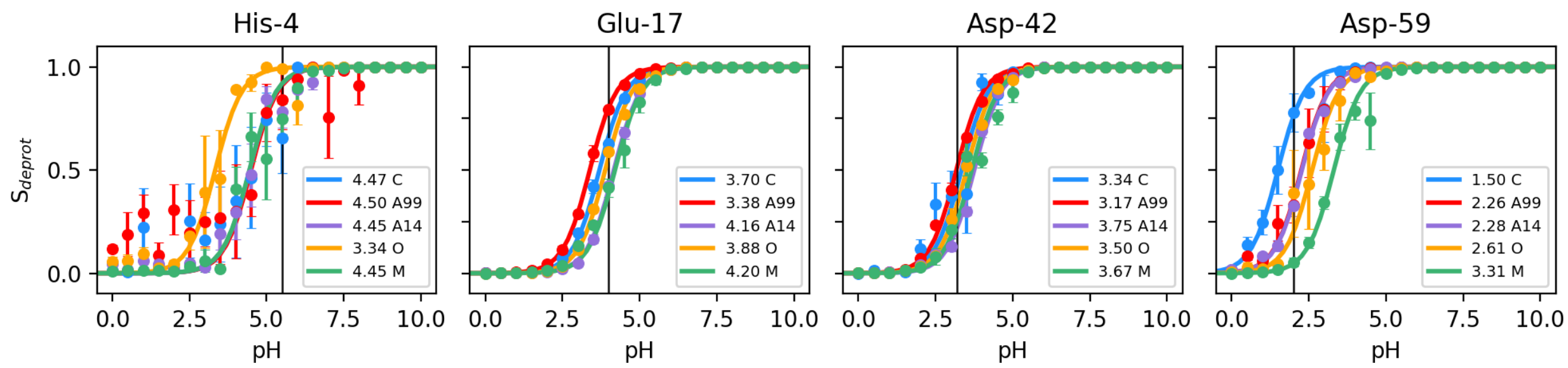

Figure S5: Titration curves for all titratable Asp, Glu and His in the cardV protein, using the CHARMM36 (blue), Amber99sb-ildn (red), Amber14sb (purple), OPLS-AA/M (yellow), and Martini 2.0 (green) force fields. The experimentally obtained pK_a values[6,7] are illustrated with vertical black lines. The estimated pK_a values are given in the legends, and the values with errors are listed in Table S2.

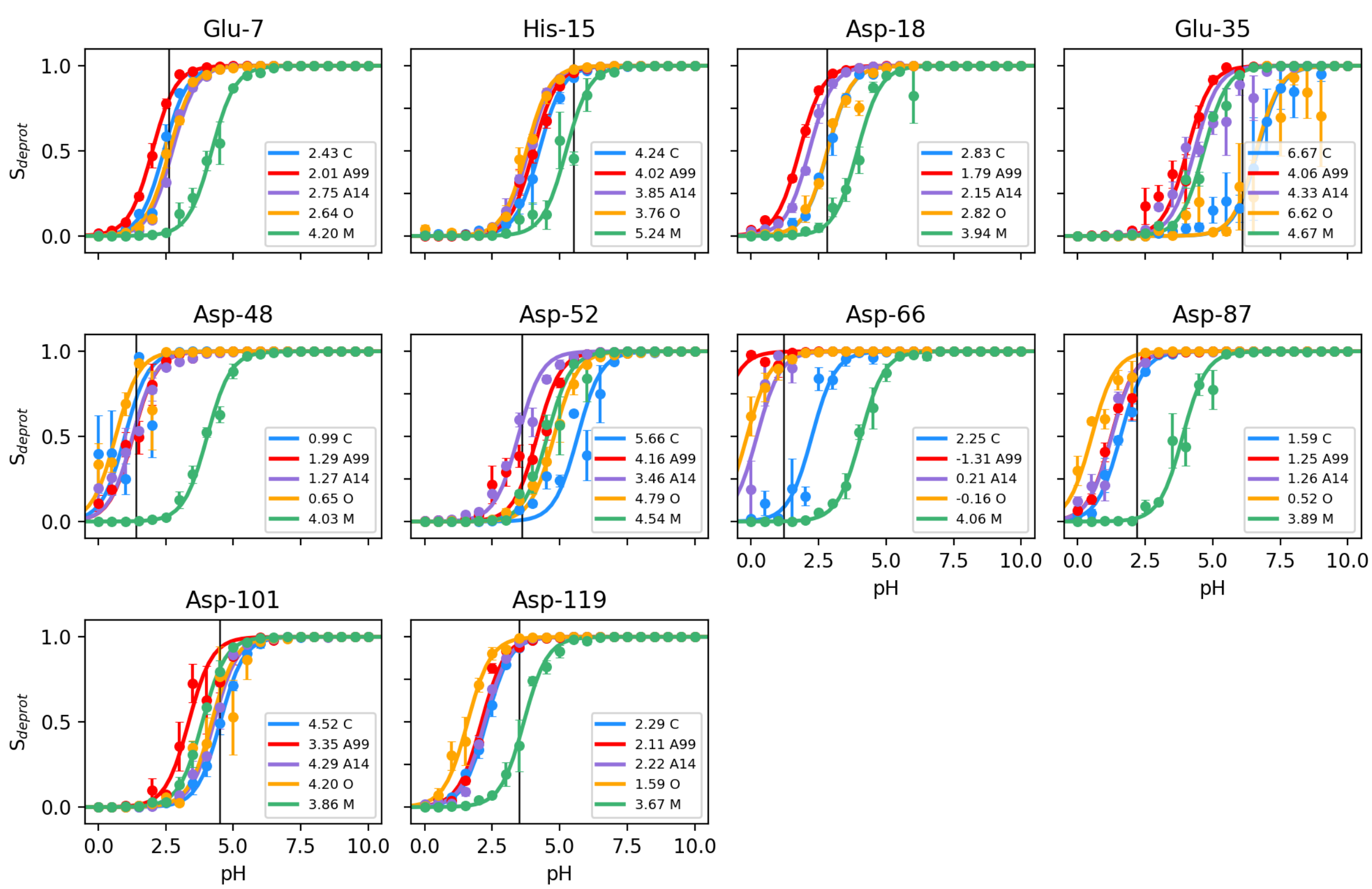

Figure S6: Titration curves for all titratable Asp, Glu and His in the HEWL protein, using the CHARMM36 (blue), Amber99sb-ildn (red), Amber14sb (purple), OPLS-AA/M (yellow), and Martini 2.0 (green) force fields. The experimentally obtained pK_a values[8] are illustrated with vertical black lines. The estimated pK_a values are given in the legends, and the values with errors are listed in Table S3.

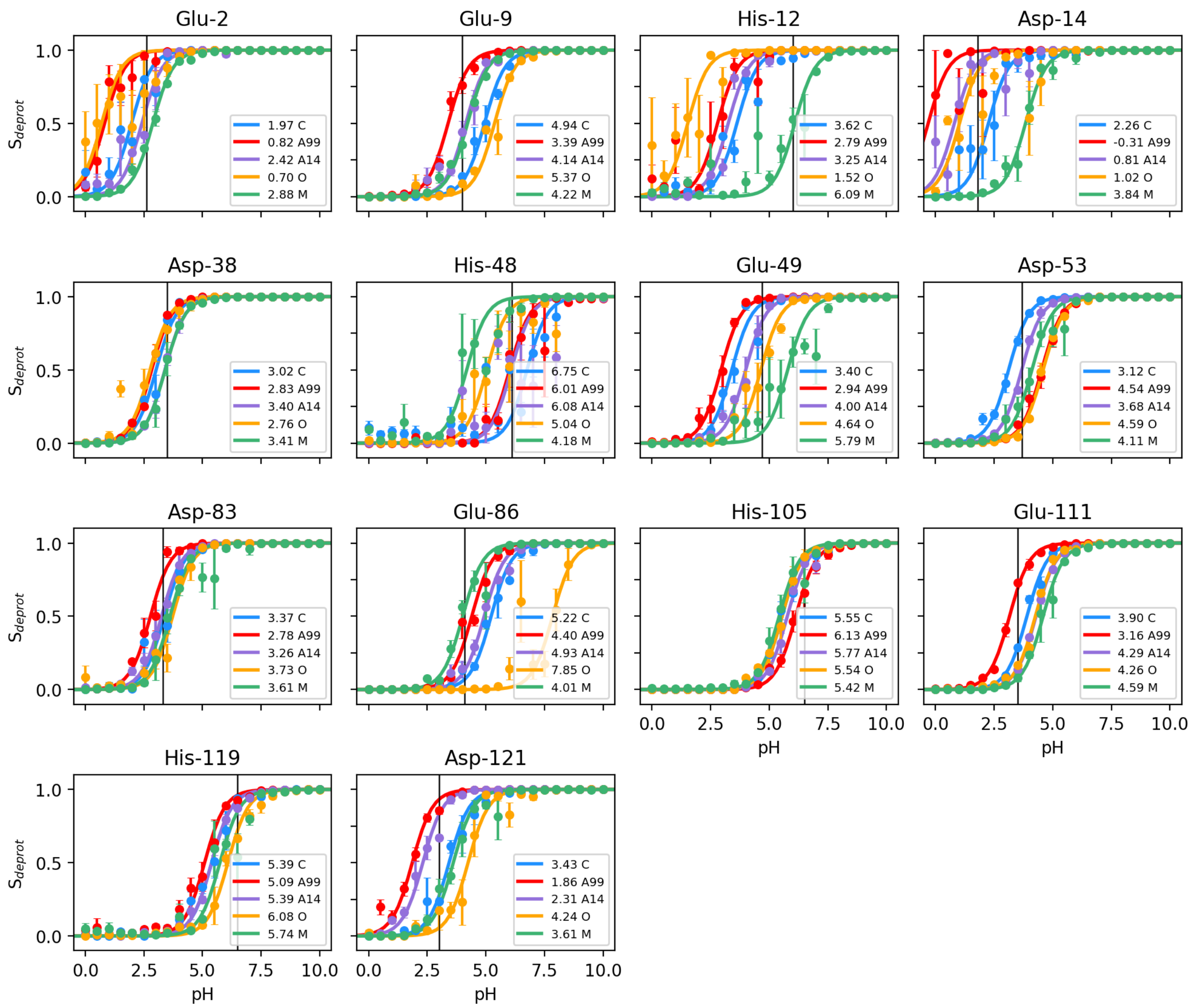

Figure S7: Titration curves for all titratable Asp, Glu and His in the RNaseA protein, using the CHARMM36 (blue), Amber99sb-ildn (red), Amber14sb (purple), OPLS-AA/M (yellow), and Martini 2.0 (green) force fields. The experimentally obtained pK_a values[9] are illustrated with vertical black lines. The estimated pK_a values are given in the legends, and the values with errors are listed in Table S4.

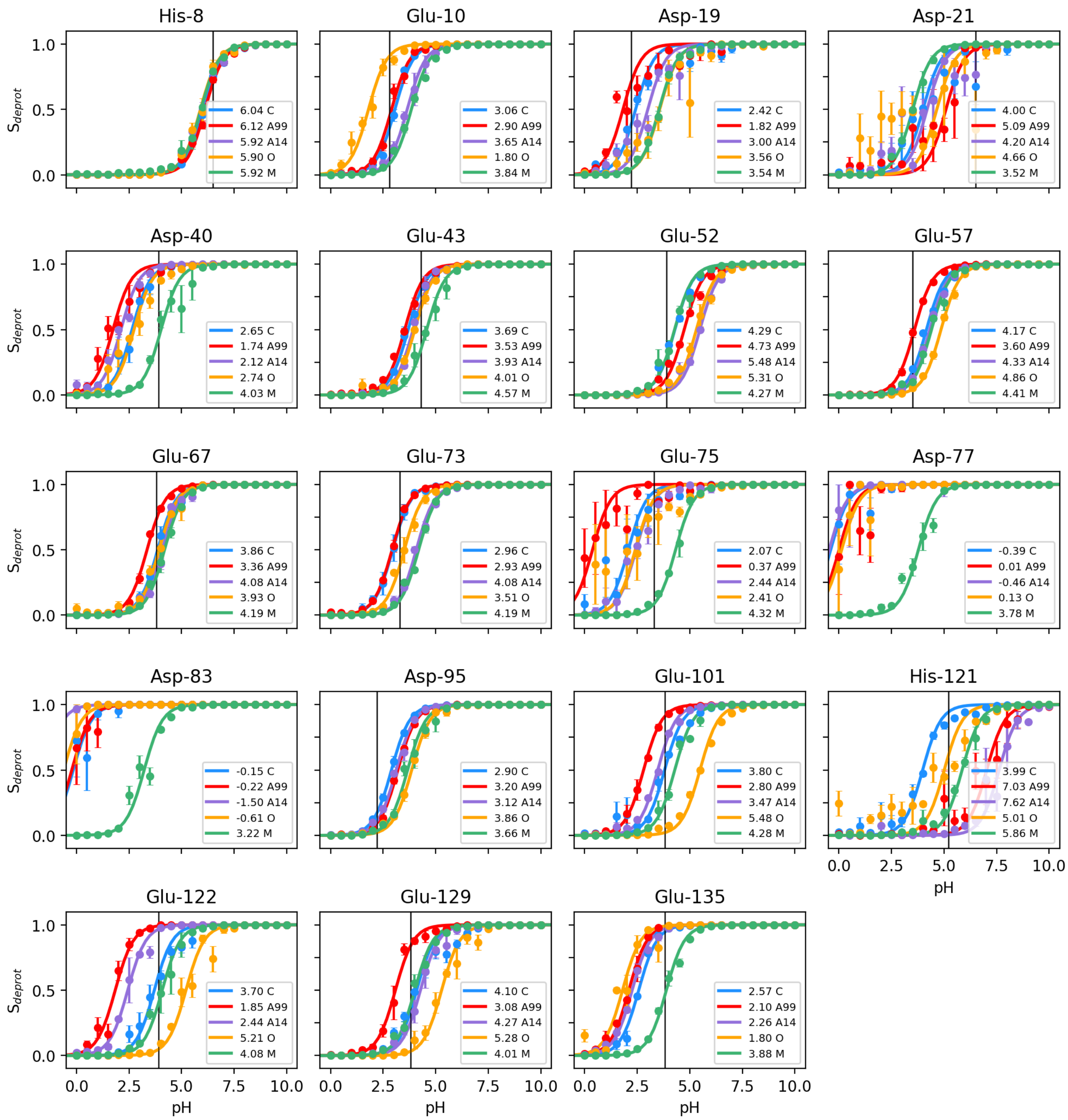

Figure S8: Titration curves for all titratable Asp, Glu and His in the SNase protein, using the CHARMM36 (blue), Amber99sb-ildn (red), Amber14sb (purple), OPLS-AA/M (yellow), and Martini 2.0 (green) force fields. The experimentally obtained pK_a values[10] are illustrated with vertical black lines. The estimated pK_a values are given in the legends, and the values with errors are listed in Table S5.

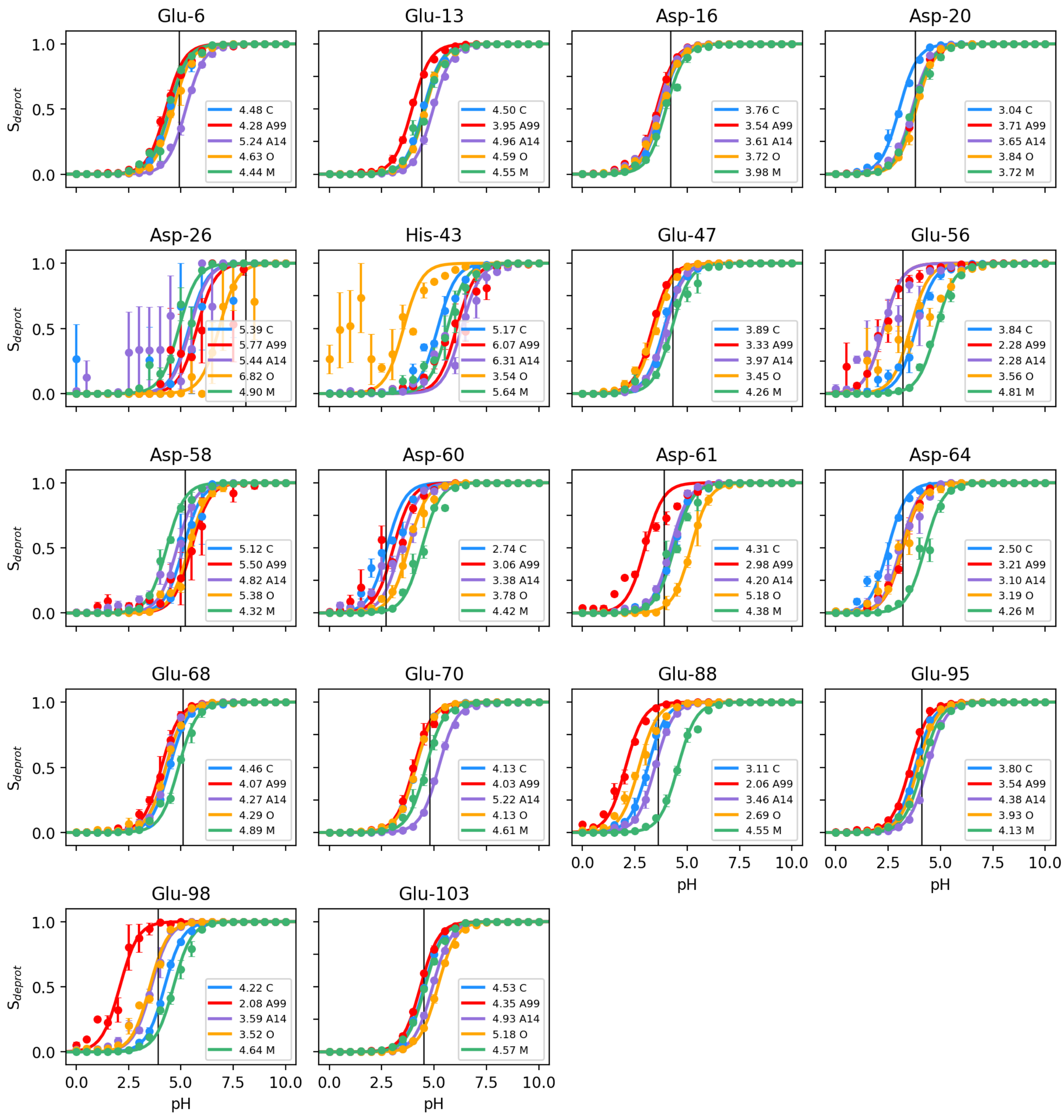

Figure S9: Titration curves for all titratable Asp, Glu and His in the thioredoxin protein, using the CHARMM36 (blue), Amber99sb-ildn (red), Amber14sb (purple), OPLS-AA/M (yellow), and Martini 2.0 (green) force fields. The experimentally obtained pK_a values[11] are illustrated with vertical black lines. The estimated pK_a values are given in the legends, and the values with errors are listed in Table S6.

residue	exp.	CHARMM36	dev.	Amber99sb-ildn	dev.	Amber14sb	dev.	OPLS-AA/M	dev.	Martini 2.0	dev.
Asp-129	3.9	2.14 ± 0.14	**-1.76**	2.70 ± 0.09	**-1.20**	3.22 ± 0.11	-0.68	2.19 ± 0.34	**-1.71**	3.46 ± 0.05	-0.44
Glu-141	4.5	4.32 ± 0.07	-0.18	3.76 ± 0.03	-0.74	4.48 ± 0.04	-0.02	4.36 ± 0.05	-0.14	4.19 ± 0.04	-0.31
His-142	6.5	5.66 ± 0.08	-0.84	5.91 ± 0.10	-0.59	5.79 ± 0.09	-0.71	5.59 ± 0.06	-0.91	6.12 ± 0.05	-0.38
Asp-145	3.7	2.66 ± 0.02	**-1.04**	2.46 ± 0.03	**-1.24**	2.44 ± 0.04	**-1.26**	2.20 ± 0.07	**-1.50**	3.61 ± 0.03	-0.09
Glu-161	3.7	3.81 ± 0.02	0.11	3.68 ± 0.04	-0.02	4.54 ± 0.03	0.84	3.75 ± 0.08	0.05	4.27 ± 0.06	0.57
Asp-162	3.2	1.01 ± 0.28	**-2.19**	2.65 ± 0.16	-0.55	1.45 ± 0.16	**-1.75**	2.73 ± 0.15	-0.47	3.61 ± 0.07	0.41
Glu-164	4.5	3.71 ± 0.08	-0.79	3.41 ± 0.07	**-1.09**	4.57 ± 0.04	0.07	3.19 ± 0.11	**-1.31**	4.07 ± 0.05	-0.43
His-166	5.4	4.65 ± 0.11	-0.75	5.49 ± 0.07	0.09	4.38 ± 0.06	**-1.02**	4.73 ± 0.08	-0.67	6.34 ± 0.05	0.94
RMSE		1.17		0.82		0.96		1.03		0.50	

Table S1: Calculated pK_a values for BBL using the CHARMM36, Amber99sb-ildn, Amber14sb, OPLS-AA/M and Martini 2.0 force fields, compared to the experimentally obtained values in the second column.[4,5] The columns titled dev. after each force field give the deviation of the calculated value from the experimental one, and deviations >1 are highlighted in bold.

residue	exp.	CHARMM36	dev.	Amber99sb-ildn	dev.	Amber14sb	dev.	OPLS-AA/M	dev.	Martini 2.0	dev.
His-4	5.5	4.47 ± 0.24	**-1.03**	4.50 ± 0.33	**-1.00**	4.45 ± 0.12	**-1.05**	3.34 ± 0.14	**-2.16**	4.45 ± 0.18	**-1.05**
Glu-17	4.0	3.70 ± 0.03	-0.30	3.38 ± 0.01	-0.62	4.16 ± 0.01	0.16	3.88 ± 0.03	-0.12	4.20 ± 0.07	0.20
Asp-42	3.2	3.34 ± 0.17	0.14	3.17 ± 0.04	-0.03	3.75 ± 0.05	0.55	3.50 ± 0.03	0.30	3.67 ± 0.13	0.47
Asp-59	2.0	1.50 ± 0.09	-0.50	2.26 ± 0.06	0.26	2.28 ± 0.05	0.28	2.61 ± 0.12	0.61	3.31 ± 0.11	**1.31**
RMSE		0.60		0.60		0.61		1.14		0.88	

Table S2: Calculated pK_a values for cardiotoxin V using the CHARMM36, Amber99sb-ildn, Amber14sb, OPLS-AA/M and Martini 2.0 force fields, compared to the experimentally obtained values in the second column.[6,7] The columns titled dev. after each force field give the deviation of the calculated value from the experimental one, and deviations >1 are highlighted in bold.

residue	exp.	CHARMM36	dev.	Amber99sb-ildn	dev.	Amber14sb	dev.	OPLS-AA/M	dev.	Martini 2.0	dev.
Glu-7	2.6	2.43 ± 0.08	-0.17	2.01 ± 0.03	-0.59	2.75 ± 0.06	0.15	2.64 ± 0.06	0.04	4.20 ± 0.08	**1.60**
His-15	5.5	4.24 ± 0.04	**-1.26**	4.02 ± 0.06	**-1.48**	3.85 ± 0.03	**-1.65**	3.76 ± 0.07	**-1.74**	5.24 ± 0.15	-0.26
Asp-18	2.8	2.83 ± 0.03	0.03	1.79 ± 0.04	**-1.01**	2.15 ± 0.03	-0.65	2.82 ± 0.10	0.02	3.94 ± 0.11	**1.14**
Glu-35	6.1	6.67 ± 0.12	0.57	4.06 ± 0.15	**-2.04**	4.33 ± 0.23	**-1.77**	6.62 ± 0.29	0.52	4.67 ± 0.10	**-1.43**
Asp-48	1.4	0.99 ± 0.29	-0.41	1.29 ± 0.10	-0.11	1.27 ± 0.12	-0.13	0.65 ± 0.18	-0.75	4.03 ± 0.07	**2.63**
Asp-52	3.6	5.66 ± 0.23	**2.06**	4.16 ± 0.20	0.56	3.46 ± 0.12	-0.14	4.79 ± 0.06	**1.19**	4.54 ± 0.13	0.94
Asp-66	1.2	2.25 ± 0.16	**1.05**	<0	>1.2	0.21 ± 0.15	-0.99	<0	-	4.06 ± 0.05	**2.86**
Asp-87	2.2	1.59 ± 0.06	-0.61	1.25 ± 0.07	**-0.95**	1.26 ± 0.10	-0.94	0.52 ± 0.12	**-1.68**	3.89 ± 0.14	**1.69**
Asp-101	4.5	4.52 ± 0.04	0.02	3.35 ± 0.17	**-1.15**	4.29 ± 0.05	-0.21	4.20 ± 0.20	-0.30	3.86 ± 0.02	-0.64
Asp-119	3.5	2.29 ± 0.03	**-1.21**	2.11 ± 0.07	**-1.39**	2.22 ± 0.05	**-1.28**	1.59 ± 0.06	**-1.91**	3.67 ± 0.05	0.17
RMSE		0.97		1.17		0.99		1.15		1.59	

Table S3: Calculated pK_a values for HEWL using the CHARMM36, Amber99sb-ildn, Amber14sb, OPLS-AA/M and Martini 2.0 force fields, compared to the experimentally obtained values in the second column.[8] The columns titled dev. after each force field give the deviation of the calculated value from the experimental one, and deviations >1 are highlighted in bold.

residue	exp.	CHARMM36	dev.	Amber99sb-ildn	dev.	Amber14sb	dev.	OPLS-AA/M	dev.	Martini 2.0	dev.
Glu-2	2.6	1.97 ± 0.18	-0.63	0.82 ± 0.14	**-1.78**	2.42 ± 0.17	-0.18	0.70 ± 0.34	**-1.90**	2.88 ± 0.05	0.28
Glu-9	4.0	4.94 ± 0.08	0.94	3.39 ± 0.06	-0.61	4.14 ± 0.10	0.14	5.37 ± 0.13	**1.37**	4.22 ± 0.05	0.22
His-12	6.0	3.62 ± 0.19	**-2.38**	2.79 ± 0.23	**-3.21**	3.25 ± 0.12	**-2.75**	1.52 ± 0.27	**-4.48**	6.09 ± 0.28	0.09
Asp-14	1.8	2.26 ± 0.18	0.46	<0	>1.8	0.81 ± 0.22	-0.99	1.02 ± 0.35	-0.78	3.84 ± 0.08	**2.04**
Asp-38	3.5	3.02 ± 0.10	-0.48	2.83 ± 0.05	-0.67	3.40 ± 0.04	-0.10	2.76 ± 0.17	-0.74	3.41 ± 0.03	-0.09
His-48	6.1	6.75 ± 0.35	0.65	6.01 ± 0.19	-0.09	6.08 ± 0.47	-0.02	5.04 ± 0.28	**-1.06**	4.18 ± 0.26	**-1.92**
Glu-49	4.7	3.40 ± 0.14	**-1.30**	2.94 ± 0.05	**-1.76**	4.00 ± 0.07	-0.70	4.64 ± 0.13	-0.06	5.79 ± 0.26	**1.09**
Asp-53	3.7	3.12 ± 0.05	-0.58	4.54 ± 0.05	0.84	3.68 ± 0.03	-0.02	4.59 ± 0.03	0.89	4.11 ± 0.12	0.41
Asp-83	3.3	3.37 ± 0.13	0.07	2.78 ± 0.09	-0.52	3.26 ± 0.06	-0.04	3.73 ± 0.12	0.43	3.61 ± 0.16	0.31
Glu-86	4.1	5.22 ± 0.08	**1.12**	4.40 ± 0.10	0.30	4.93 ± 0.07	0.83	7.85 ± 0.30	**3.75**	4.01 ± 0.14	-0.09
His-105	6.5	5.55 ± 0.06	-0.95	6.13 ± 0.06	-0.37	5.77 ± 0.06	-0.73	5.54 ± 0.05	**-0.96**	5.42 ± 0.11	**-1.08**
Glu-111	3.5	3.90 ± 0.05	0.40	3.16 ± 0.03	-0.34	4.29 ± 0.02	0.79	4.26 ± 0.05	0.76	4.59 ± 0.07	**1.09**
His-119	6.5	5.39 ± 0.10	**-1.11**	5.09 ± 0.11	**-1.41**	5.39 ± 0.06	**-1.11**	6.08 ± 0.08	-0.42	5.74 ± 0.22	-0.76
Asp-121	3.0	3.43 ± 0.11	0.43	1.86 ± 0.09	**-1.14**	2.31 ± 0.10	0.69	4.24 ± 0.14	**1.24**	3.61 ± 0.12	0.61
RMSE		0.98		1.30		0.95		1.81		0.95	

Table S4: Calculated pK_a values for RNase A using the CHARMM36, Amber99sb-ildn, Amber14sb, OPLS-AA/M and Martini 2.0 force fields, compared to the experimental values in the second column.[9] The columns titled dev. after each force field give the deviation of the calculated value from the experimental one, and deviations >1 are highlighted in bold.

residue	exp.	CHARMM36	dev.	Amber99sb-ildn	dev.	Amber14sb	dev.	OPLS-AA/M	dev.	Martini 2.0	dev.
His-8	6.5	6.04 ± 0.04	-0.46	6.12 ± 0.05	-0.38	5.92 ± 0.06	-0.58	5.90 ± 0.06	-0.60	5.92 ± 0.05	-0.58
Glu-10	2.8	3.06 ± 0.05	0.26	2.90 ± 0.06	0.10	3.65 ± 0.04	0.85	1.80 ± 0.09	-1.00	3.84 ± 0.06	**1.04**
Asp-19	2.2	2.42 ± 0.17	0.22	1.82 ± 0.23	-0.38	3.00 ± 0.20	0.80	3.56 ± 0.25	**1.36**	3.54 ± 0.04	**1.34**
Asp-21	6.5	4.00 ± 0.28	**-2.50**	5.09 ± 0.25	**-1.41**	4.20 ± 0.32	**-2.30**	4.66 ± 0.57	**-1.84**	3.52 ± 0.04	**-2.98**
Asp-40	3.9	2.65 ± 0.08	**-1.25**	1.74 ± 0.15	**-2.16**	2.12 ± 0.09	**-1.78**	2.74 ± 0.15	**-1.16**	4.03 ± 0.15	0.13
Glu-43	4.3	3.69 ± 0.05	-0.61	3.53 ± 0.06	-0.77	3.93 ± 0.04	-0.37	4.01 ± 0.04	-0.29	4.57 ± 0.07	0.27
Glu-52	3.9	4.29 ± 0.06	0.39	4.73 ± 0.08	0.83	5.48 ± 0.06	**1.58**	5.31 ± 0.07	**1.41**	4.27 ± 0.08	0.37
Glu-57	3.5	4.17 ± 0.07	0.67	3.60 ± 0.03	0.10	4.33 ± 0.04	0.83	4.86 ± 0.03	**1.36**	4.41 ± 0.06	0.91
Glu-67	3.8	3.86 ± 0.04	0.06	3.36 ± 0.03	-0.44	4.08 ± 0.08	0.28	3.93 ± 0.08	0.13	4.19 ± 0.05	0.39
Glu-73	3.3	2.96 ± 0.02	-0.34	2.93 ± 0.02	-0.37	4.08 ± 0.05	0.78	3.51 ± 0.06	0.21	4.19 ± 0.05	**0.89**
Glu-75	3.3	2.07 ± 0.21	-1.23	0.37 ± 0.22	**-2.93**	2.44 ± 0.12	-0.86	2.41 ± 0.32	-0.89	4.32 ± 0.04	**1.02**
Asp-77	2.2	<0	>2.2	0.01 ± 0.30	**-2.19**	<0	>2.2	0.13 ± 0.16	**-2.07**	3.78 ± 0.11	**1.58**
Asp-83	2.2	<0	>2.2	<0	>2.2	<0	>2.2	<0	>2.2	3.22 ± 0.15	**1.02**
Asp-95	2.2	2.90 ± 0.01	0.70	3.20 ± 0.03	1.00	3.12 ± 0.04	0.92	3.86 ± 0.04	**1.66**	3.66 ± 0.08	**1.46**
Glu-101	3.8	3.80 ± 0.13	0.00	2.80 ± 0.02	-1.00	3.47 ± 0.05	-0.33	5.48 ± 0.07	**1.68**	4.28 ± 0.08	0.48
His-121	5.2	3.99 ± 0.12	**-1.21**	7.03 ± 0.22	**1.83**	7.62 ± 0.15	**2.42**	5.01 ± 0.33	-0.19	5.86 ± 0.08	0.66
Glu-122	3.9	3.70 ± 0.12	-0.20	1.85 ± 0.09	**-2.05**	2.44 ± 0.07	**-1.46**	5.21 ± 0.14	**1.31**	4.08 ± 0.08	0.18
Glu-129	3.8	4.10 ± 0.15	0.30	3.08 ± 0.06	-0.72	4.27 ± 0.07	0.47	5.28 ± 0.10	**1.48**	4.01 ± 0.06	0.21
Glu-135	3.8	2.57 ± 0.05	**-1.23**	2.10 ± 0.05	**-1.70**	2.26 ± 0.04	**-1.54**	1.80 ± 0.15	**-2.00**	3.88 ± 0.06	0.08
RMSE		0.92		1.33		1.25		1.24		1.03	

Table S5: Calculated pK_a values for SNase using the CHARMM36, Amber99sb-ildn, Amber14sb, OPLS-AA/M and Martini 2.0 force fields, compared to the experimental values in the second column.[10] The columns titled dev. after each force field give the deviation of the calculated value from the experimental one, and deviations >1 are highlighted in bold.

residue	exp.	CHARMM36	dev.	Amber99sb-ildn	dev.	Amber14sb	dev.	OPLS-AA/M	dev.	Martini 2.0	dev.
Glu-6	4.9	4.48 ± 0.05	-0.42	4.28 ± 0.07	**-0.62**	5.24 ± 0.03	0.34	4.63 ± 0.05	-0.27	4.44 ± 0.07	-0.46
Glu-13	4.4	4.50 ± 0.03	0.10	3.95 ± 0.03	-0.45	4.96 ± 0.03	0.56	4.59 ± 0.03	0.19	4.55 ± 0.08	0.15
Asp-16	4.2	3.76 ± 0.03	-0.44	3.54 ± 0.04	-0.66	3.61 ± 0.05	-0.59	3.72 ± 0.05	-0.48	3.98 ± 0.06	-0.22
Asp-20	3.8	3.04 ± 0.05	-0.76	3.71 ± 0.02	-0.09	3.65 ± 0.04	-0.15	3.84 ± 0.03	0.04	3.72 ± 0.06	-0.08
Asp-26	8.1	5.39 ± 0.33	**-2.71**	5.77 ± 0.30	**-2.33**	5.44 ± 0.46	**-2.66**	6.82 ± 0.16	**-1.28**	4.90 ± 0.17	**-3.20**
His-43	n/a	5.17 ± 0.14	n/a	6.07 ± 0.22	n/a	6.31 ± 0.23	n/a	3.54 ± 0.57	n/a	5.64 ± 0.11	n/a
Glu-47	4.3	3.89 ± 0.05	-0.41	3.33 ± 0.02	-0.97	3.97 ± 0.03	-0.33	3.45 ± 0.06	-0.85	4.26 ± 0.08	-0.04
Glu-56	3.2	3.84 ± 0.09	0.64	2.28 ± 0.13	-0.92	2.28 ± 0.28	-0.92	3.56 ± 0.35	0.36	4.81 ± 0.06	**1.61**
Asp-58	5.2	5.12 ± 0.11	-0.08	5.50 ± 0.13	0.30	4.82 ± 0.21	-0.38	5.38 ± 0.08	0.18	4.32 ± 0.06	-0.88
Asp-60	2.7	2.74 ± 0.18	0.04	3.06 ± 0.26	0.36	3.38 ± 0.18	0.68	3.78 ± 0.06	**1.08**	4.42 ± 0.09	**1.72**
Asp-61	3.9	4.31 ± 0.03	0.41	2.98 ± 0.18	-0.92	4.20 ± 0.05	0.30	5.18 ± 0.02	**1.28**	4.38 ± 0.10	0.48
Asp-64	3.2	2.50 ± 0.10	-0.70	3.21 ± 0.05	0.01	3.10 ± 0.10	-0.10	3.19 ± 0.11	-0.01	4.26 ± 0.11	**1.06**
Glu-68	5.1	4.46 ± 0.03	-0.64	4.07 ± 0.04	**-1.03**	4.27 ± 0.05	-0.83	4.29 ± 0.04	-0.81	4.89 ± 0.07	-0.21
Glu-70	4.8	4.13 ± 0.02	-0.67	4.03 ± 0.04	-0.77	5.33 ± 0.02	0.42	4.13 ± 0.02	-0.67	4.61 ± 0.05	-0.19
Glu-88	3.6	3.11 ± 0.04	-0.49	2.06 ± 0.07	**-1.54**	3.46 ± 0.02	-0.14	2.69 ± 0.09	-0.91	4.55 ± 0.06	0.95
Glu-95	4.1	3.80 ± 0.02	-0.30	3.54 ± 0.03	-0.56	4.38 ± 0.03	0.28	3.93 ± 0.03	-0.17	4.13 ± 0.05	0.03
Glu-98	3.9	4.22 ± 0.02	0.32	2.08 ± 0.12	**-1.82**	3.59 ± 0.08	+0.31	3.52 ± 0.10	-0.38	4.64 ± 0.09	0.74
Glu-103	4.5	4.53 ± 0.02	0.03	4.35 ± 0.02	-0.15	4.93 ± 0.02	0.43	5.18 ± 0.03	0.68	4.57 ± 0.03	0.07
RMSE		0.80		1.00		0.90		0.70		1.08	

Table S6: Calculated pK_a values for thioredoxin using the CHARMM36, Amber99sb-ildn, Amber14sb, OPLS-AA/M and Martini 2.0 force fields, compared to the experimental values in the second column.[11] The columns titled dev. after each force field give the deviation of the calculated value from the experimental one, and deviations >1 are highlighted in bold.

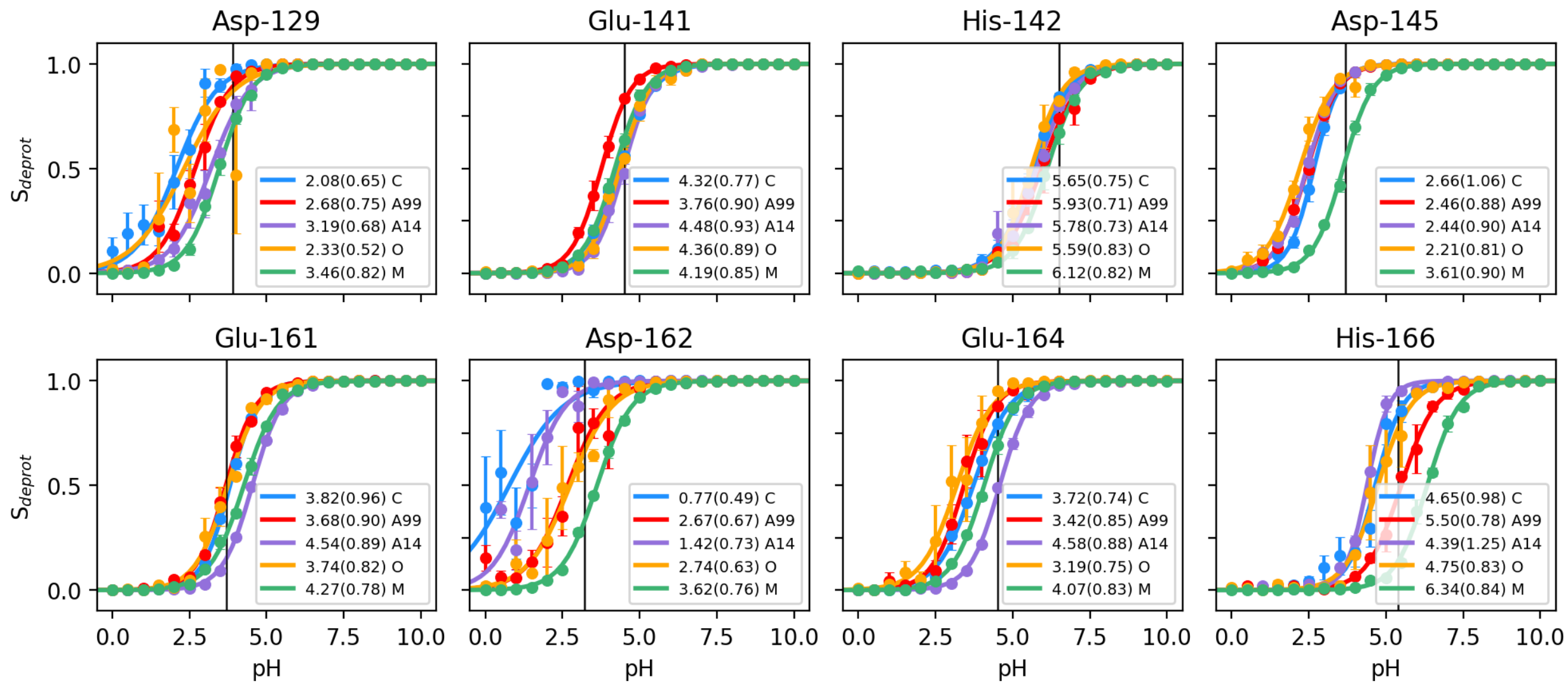

Figure S10: Titration curves fitted to the Hill equation for all titratable Asp, Glu and His in the BBL protein, using the CHARMM36 (blue), Amber99sb-ildn (red), Amber14sb (purple), OPLS-AA/M (yellow), and Martini 2.0 (green) force fields. The experimentally obtained pK_a values[4,5] are illustrated with vertical black lines. The estimated pK_a values and the Hill coefficients n in brackets are given in the legends.

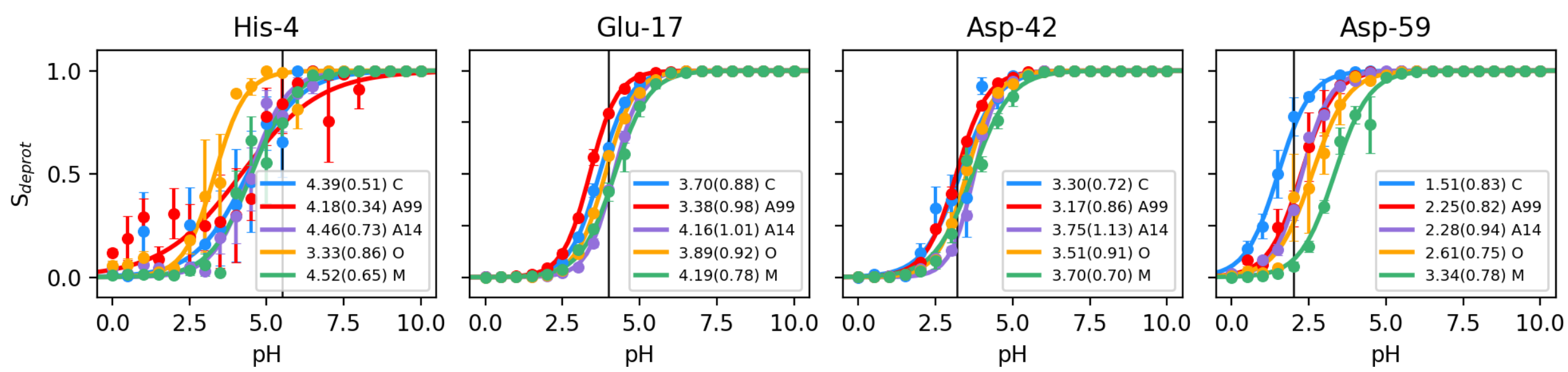

Figure S11: Titration curves fitted to the Hill equation for all titratable Asp, Glu and His in the cardV protein, using the CHARMM36 (blue), Amber99sb-ildn (red), Amber14sb (purple), OPLS-AA/M (yellow), and Martini 2.0 (green) force fields. The experimentally obtained pK_a values[6,7] are illustrated with vertical black lines. The estimated pK_a values and the Hill coefficients n in brackets are given in the legends.

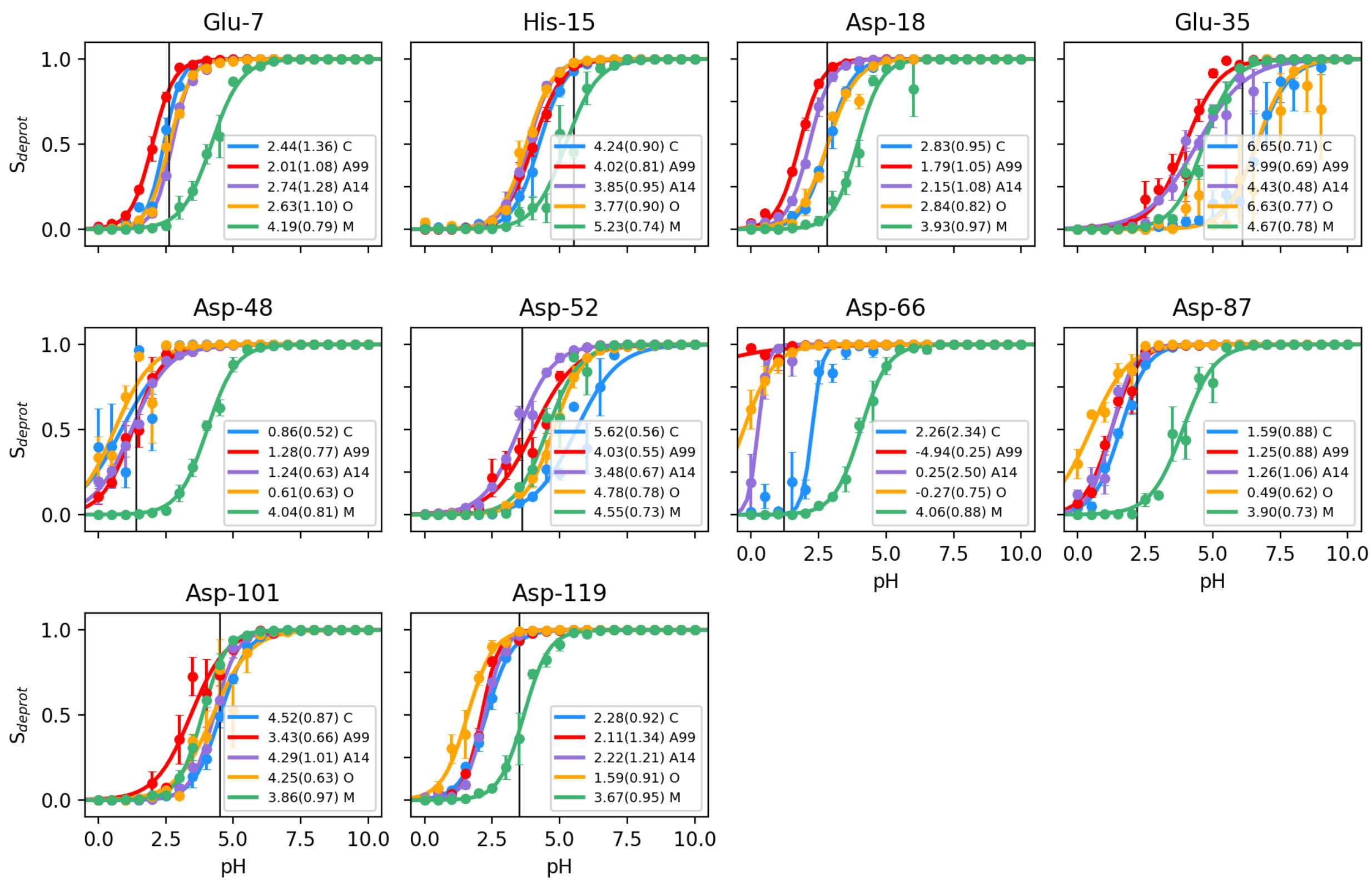

Figure S12: Titration curves fitted to the Hill equation for all titratable Asp, Glu and His in the HEWL protein, using the CHARMM36 (blue), Amber99sb-ildn (red), Amber14sb (purple), OPLS-AA/M (yellow), and Martini 2.0 (green) force fields. The experimentally obtained pK_a values[8] are illustrated with vertical black lines. The estimated pK_a values and the Hill coefficients n in brackets are given in the legends.

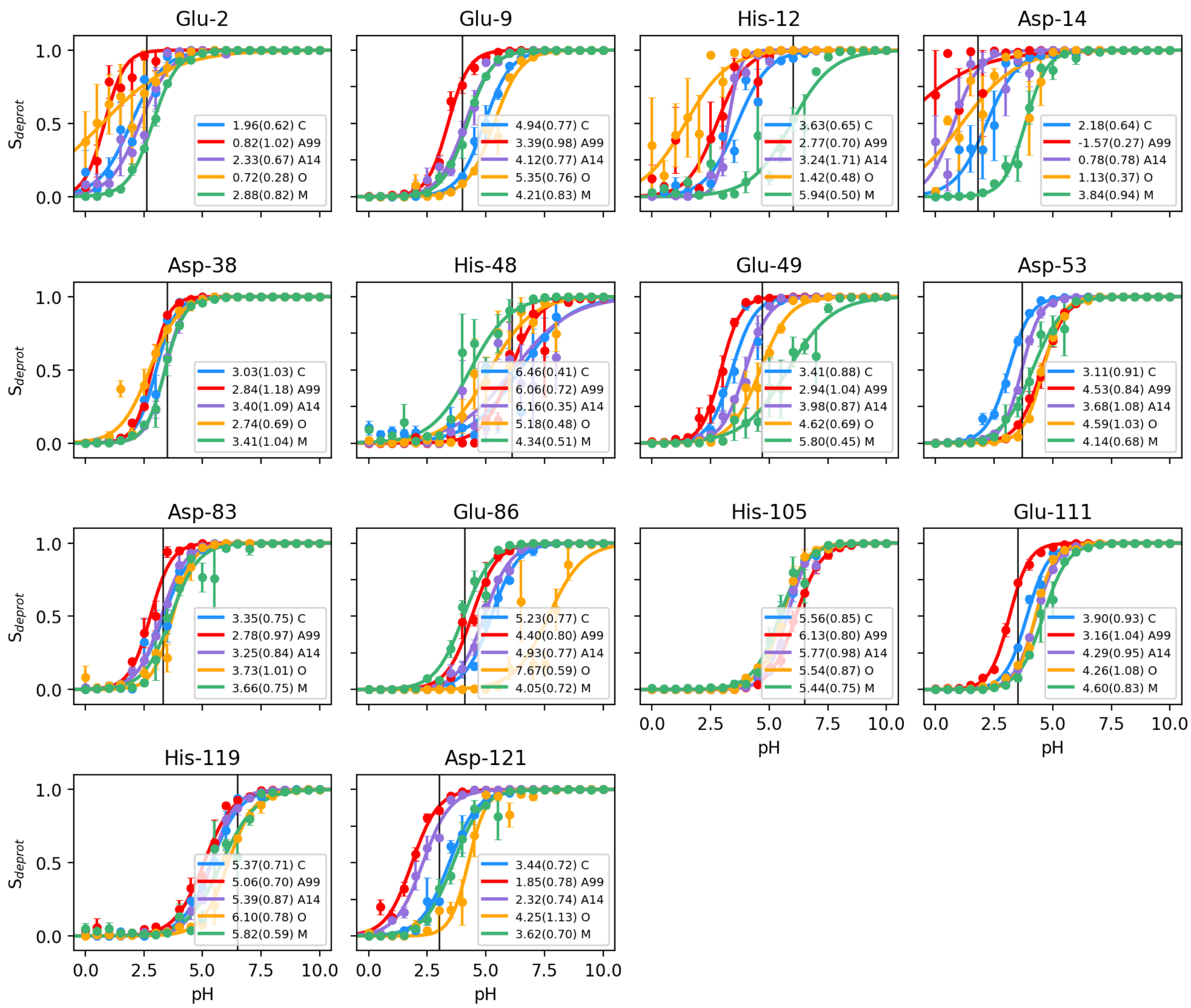

Figure S13: Titration curves fitted to the Hill equation for all titratable Asp, Glu and His in the RNaseA protein, using the CHARMM36 (blue), Amber99sb-ildn (red), Amber14sb (purple), OPLS-AA/M (yellow), and Martini 2.0 (green) force fields. The experimentally obtained pK_{a} values[9] are illustrated with vertical black lines. The estimated pK_{a} values and the Hill coefficients n in brackets are given in the legends.

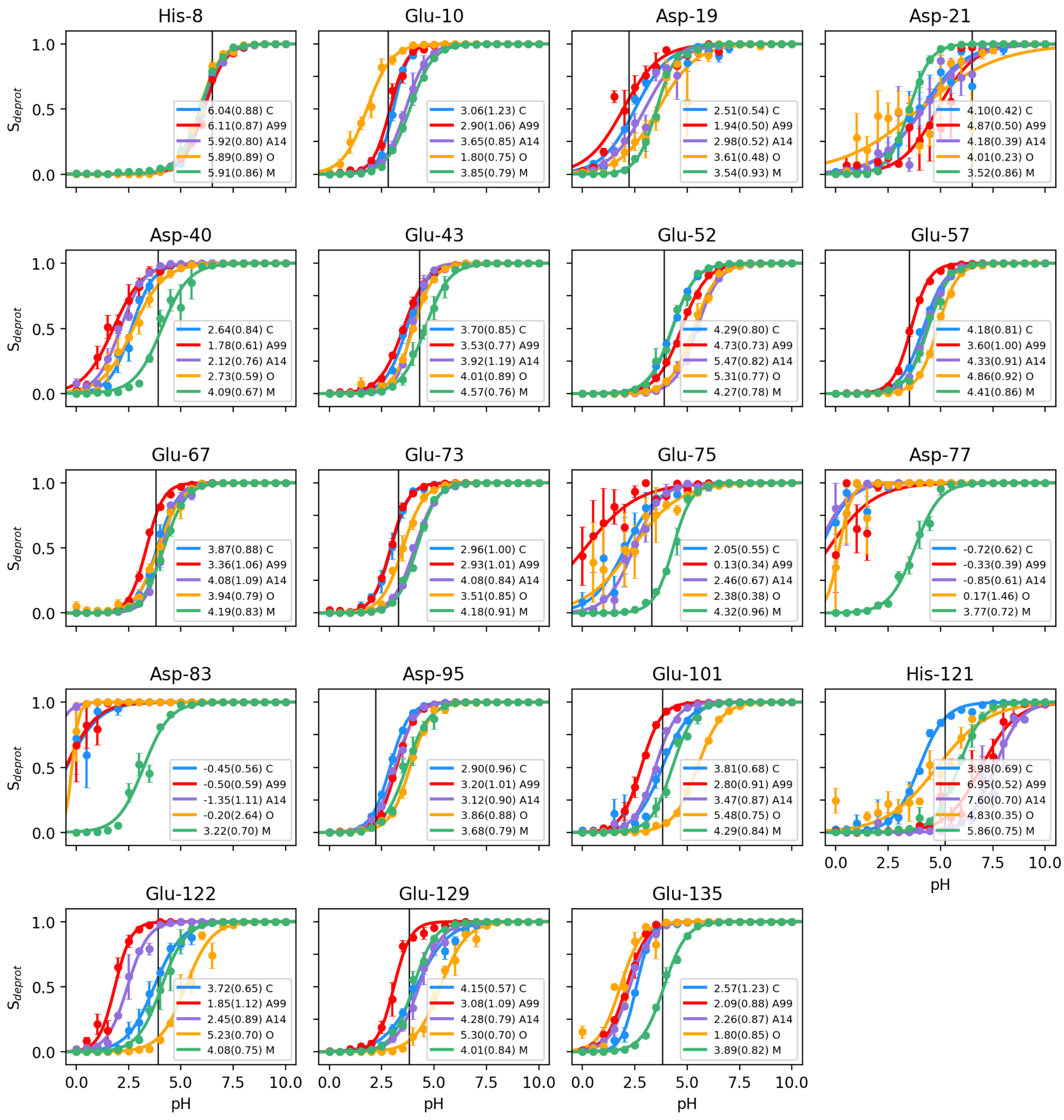

Figure S14: Titration curves fitted to the Hill equation for all titratable Asp, Glu and His in the SNase protein, using the CHARMM36 (blue), Amber99sb-ildn (red), Amber14sb (purple), OPLS-AA/M (yellow), and Martini 2.0 (green) force fields. The experimentally obtained pK_a values[10] are illustrated with vertical black lines. The estimated pK_a values and the Hill coefficients n in brackets are given in the legends.

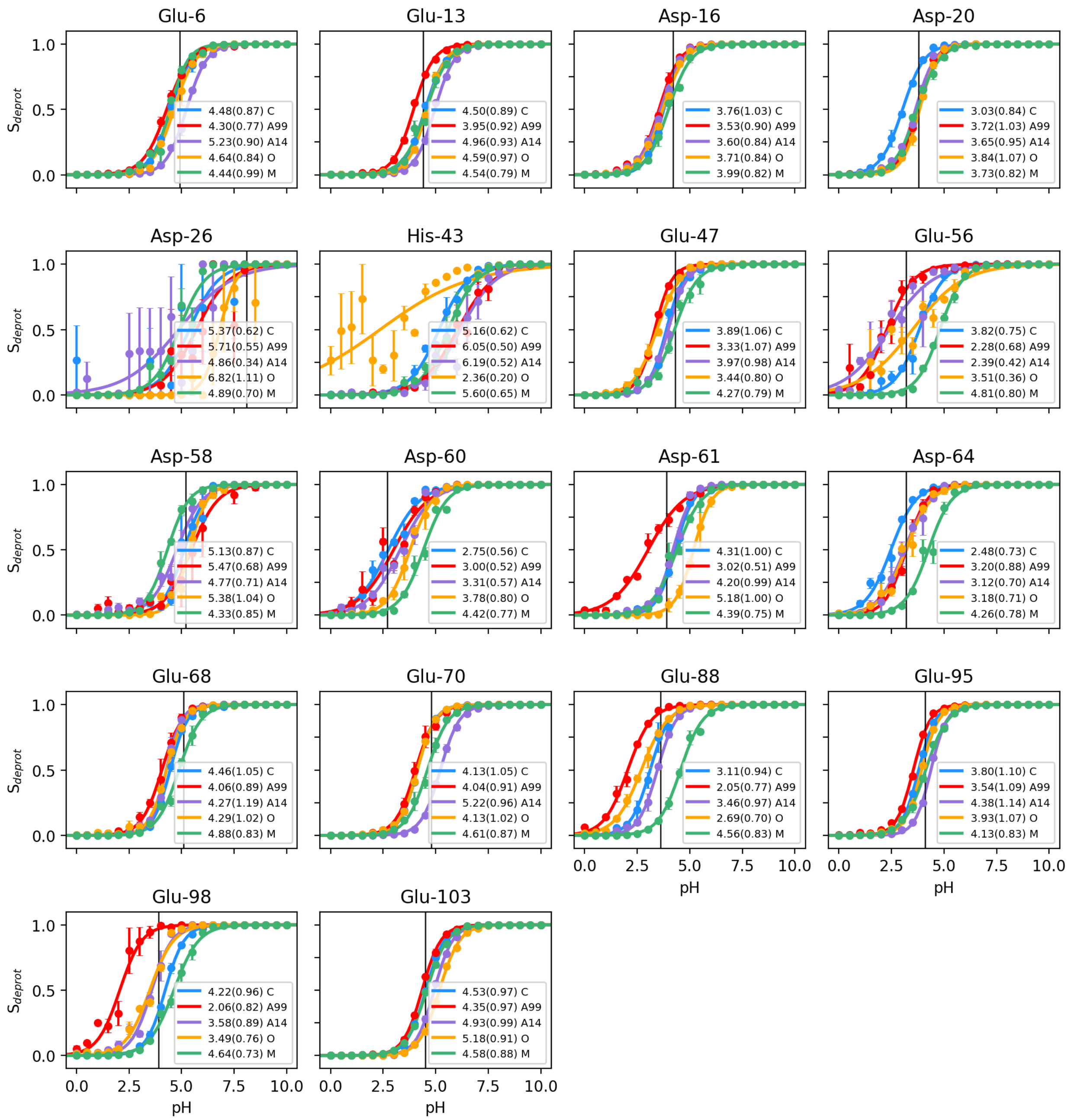

Figure S15: Titration curves fitted to the Hill equation for all titratable Asp, Glu and His in the thioredoxin protein, using the CHARMM36 (blue), Amber99sb-ildn (red), Amber14sb (purple), OPLS-AA/M (yellow), and Martini 2.0 (green) force fields. The experimentally obtained pK_a values[11] are illustrated with vertical black lines. The estimated pK_a values and the Hill coefficients n in brackets are given in the legends.

References

(1) Buslaev, P.; Aho, N.; Jansen, A.; Bauer, P.; Hess, B.; Groenhof, G. Best practices in constant pH MD simulations: accuracy and sampling. *Journal of Chemical Theory and Computation* **2022**, *18*, 6134–6147.

(2) Lindahl, V.; Lidmar, J.; Hess, B. Accelerated weight histogram method for exploring free energy landscapes. *The Journal of chemical physics* **2014**, *141*.

(3) Abraham, M. J.; Murtola, T.; Schulz, R.; Páll, S.; Smith, J. C.; Hess, B.; Lindahl, E. GROMACS: High performance molecular simulations through multi-level parallelism from laptops to supercomputers. *SoftwareX* **2015**, *1*, 19–25.

(4) Arbely, E.; Rutherford, T. J.; Sharpe, T. D.; Ferguson, N.; Fersht, A. R. Downhill versus barrier-limited folding of BBL 1: energetic and structural perturbation effects upon protonation of a histidine of unusually low pKa. *Journal of molecular biology* **2009**, *387*, 986–992.

(5) Arbely, E.; Rutherford, T. J.; Neuweiler, H.; Sharpe, T. D.; Ferguson, N.; Fersht, A. R. Carboxyl pKa values and acid denaturation of BBL. *Journal of molecular biology* **2010**, *403*, 313–327.

(6) Chiang, C.-M.; Chien, K.-Y.; Lin, H.-j.; Lin, J.-F.; Yeh, H.-C.; Ho, P.-l.; Wu, W.-g. Conformational change and inactivation of membrane phospholipid-related activity of cardiotoxin V from Taiwan cobra venom at acidic pH. *Biochemistry* **1996**, *35*, 9167–9176.

(7) Chiang, C.-M.; Chang, S.-L.; Lin, H.-j.; Wu, W.-g. The role of acidic amino acid residues in the structural stability of snake cardiotoxins. *Biochemistry* **1996**, *35*, 9177–9186.

(8) Webb, H.; Tynan-Connolly, B. M.; Lee, G. M.; Farrell, D.; O'Meara, F.; Søndergaard, C. R.; Teilum, K.; Hewage, C.; McIntosh, L. P.; Nielsen, J. E. Remeasuring

HEWL pKa values by NMR spectroscopy: Methods, analysis, accuracy, and implications for theoretical pKa calculations. *Proteins: Structure, Function, and Bioinformatics* **2011**, *79*, 685–702.

(9) Baker, W. R.; Kintanar, A. Characterization of the pH titration shifts of ribonuclease A by one-and two-dimensional nuclear magnetic resonance spectroscopy. *Archives of biochemistry and biophysics* **1996**, *327*, 189–199.

(10) Castaneda, C. A.; Fitch, C. A.; Majumdar, A.; Khangulov, V.; Schlessman, J. L.; García-Moreno, B. E. Molecular determinants of the pKa values of Asp and Glu residues in staphylococcal nuclease. *Proteins: Structure, Function, and Bioinformatics* **2009**, *77*, 570–588.

(11) Qin, J.; Clore, G. M.; Gronenborn, A. M. Ionization equilibria for side-chain carboxyl groups in oxidized and reduced human thioredoxin and in the complex with its target peptide from the transcription factor NFκB. *Biochemistry* **1996**, *35*, 7–13.
